# Metal–Support Interaction and Charge Distribution in Ceria-Supported Au Particles Exposed to CO

**DOI:** 10.1021/acs.chemmater.2c01659

**Published:** 2022-08-23

**Authors:** Oleksii Bezkrovnyi, Albert Bruix, Dominik Blaumeiser, Lesia Piliai, Simon Schötz, Tanja Bauer, Ivan Khalakhan, Tomáš Skála, Peter Matvija, Piotr Kraszkiewicz, Mirosława Pawlyta, Mykhailo Vorokhta, Iva Matolínová, Jörg Libuda, Konstantin M. Neyman, Leszek Kȩpiński

**Affiliations:** † W. Trzebiatowski Institute of Low Temperature and Structure Research, Polish Academy of Sciences, 50-422 Wroclaw, Poland; ‡ Departament de Ciència de Materials i Química Física and Institut de Química Teòrica i Computacional (IQTCUB), Universitat de Barcelona, 08028 Barcelona, Spain; § Interface Research and Catalysis, Erlangen Center for Interface Research and Catalysis, Friedrich-Alexander Universität Erlangen-Nürnberg, Egerlandstraße 3, 91058 Erlangen, Germany; ⊥ Department of Surface and Plasma Science, Faculty of Mathematics and Physics, Charles University, V Holešovičkách 2, 18000, Prague 8, Czech Republic; # Materials Research Laboratory, Silesian University of Technology, Gliwice 44-100, Poland; & ICREA (Institució Catalana de Recerca i Estudis Avançats), 08010 Barcelona, Spain

## Abstract

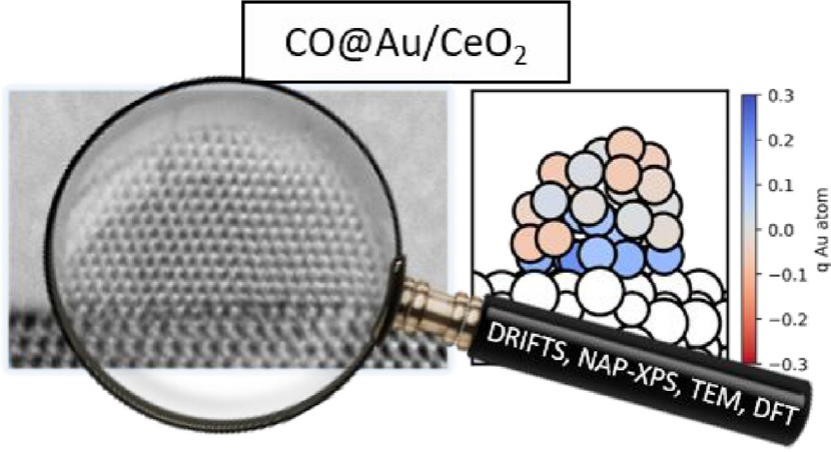

Understanding how reaction conditions affect metal–support interactions in catalytic materials is one of the most challenging tasks in heterogeneous catalysis research. Metal nanoparticles and their supports often undergo changes in structure and oxidation state when exposed to reactants, hindering a straightforward understanding of the structure–activity relations using only ex situ or ultrahigh vacuum techniques. Overcoming these limitations, we explored the metal–support interaction between gold nanoparticles and ceria supports in ultrahigh vacuum and after exposure to CO. A combination of in situ methods (on powder and model Au/CeO_2_ samples) and theoretical calculations was applied to investigate the gold/ceria interface and its reactivity toward CO exposure. X-ray photoelectron spectroscopy measurements rationalized by first-principles calculations reveal a distinctly inhomogeneous charge distribution, with Au^+^ atoms in contact with the ceria substrate and neutral Au^0^ atoms at the surface of the Au nanoparticles. Exposure to CO partially reduces the ceria substrate, leading to electron transfer to the supported Au nanoparticles. Transferred electrons can delocalize among the neutral Au atoms of the particle or contribute to forming inert Au^δ−^ atoms near oxygen vacancies at the ceria surface. This charge redistribution is consistent with the evolution of the vibrational frequencies of CO adsorbed on Au particles obtained using diffuse reflectance infrared Fourier transform spectroscopy.

## 1 Introduction

Au/ceria-based materials are well-known catalysts for low-temperature CO oxidation, which is an important reaction for the development of “green chemistry” technologies.^1^ Metal–support interactions (MSI) play a significant role in the activity of these catalysts, with the choice of support material largely defining the catalytic properties of supported Au particles.^2^ Among several other factors that control the activity of Au/ceria catalysts are (i) the size and shape of Au particles, (ii) the ability of the ceria support to uptake or supply oxygen during the reaction, (iii) the charge distribution at the metal/oxide interface determining the electronic state of the gold atoms, and (iv) changes in Au interatomic distances (strain) induced by oxide supports in different states. The size of Au particles determines the number of low-coordinated Au atoms, which are preferential sites for CO and O_2_ adsorption and, as a consequence, serve as active sites for CO oxidation reaction.^3−5^ The ability of the support to uptake or release oxygen is crucial due to the participation of ceria in CO oxidation via the Mars–van Krevelen (MvK) mechanism, which was initially proposed by Bond and Thompson^6^ and demonstrated further by various authors.^7−9^ Such a mechanism is quite common for reactions on metal particles supported by reducible oxides.^10−15^

For ceria, the O-donor/uptake behavior is strongly dependent on the predominant type of facets terminating ceria crystallites. The experimental data by Trovarelli and Llorca show that Au nanoparticles (NPs) supported by cube-shaped ceria NPs (mainly terminated by {100} facets) are more active in CO oxidation than Au NPs on ceria nanooctahedra (mainly terminated by {111} facets).^1^ This is partially related to the difference in surface diffusion of oxygen, which affects the availability of oxygen species to catalytic activity.^16^ The theoretical calculations of Castanet et al. revealed that the surface oxygen mobility on {100} surfaces of CeO_2_ is higher than on the {110} and {111} surfaces, by one and 5 orders of magnitude, respectively.^17^ In addition, the relatively high energy of oxygen vacancy formation on the CeO_2_(111) surface, in comparison to the (110) and (100) surfaces, leads to a high desorption energy of CO_2_ molecule from the Au/ceria interface, making the low-temperature CO oxidation on the gold supported by CeO_2_(111) catalyst via direct MvK mechanism almost impossible.^18^ However, the pre-existence of the oxygen vacancies, which bind O_2_ molecules,^19^ and the CO-initiated disintegration of Au NPs via the formation of isolated Au-CO complexes,^20^ may help close the second half of the MvK mechanism. Finally, some authors have attributed changes in CO oxidation activity to structural strain induced by the interaction with support,^21−23^ or to strong MSI involving the partial encapsulation of Au particles by the oxide support.^24^

In the present study, we focus on the effect of MSI on the structure and electronic state of the Au atoms, which is crucial for CO oxidation on Au/ceria catalysts. The oxidation state or electronic state of gold in the catalyst active centers is controversial and still under debate. Most Au atoms in gold-based catalysts are neutral Au^0^ species, and many authors have demonstrated that this form of gold is essential for high catalytic activity.^25,26^ However, others assigned the activity of gold catalysts to oxidized (Au^δ+^) or electron-rich (Au^δ−^) Au atoms on the perimeter of gold particles at the metal/support interface.^27,28^ Partially oxidized Au surface sites can be formed either by MSI or via chemisorption of oxygen. Shi et al. predicted the possibility of oxygen adsorption on Au at temperatures up to ∼150 °C at atmospheric pressures,^29^ and Green et al. observed chemisorption of O_2_ experimentally at perimeter sites of the Au/TiO_2_ catalyst.^30^

Recently, electron-rich Au atoms (Au^δ−^) have attracted attention in the literature. Au^δ−^ sites are created via electron transfer to Au atoms at the Au/ceria interface, directly contacting oxygen vacancies.^31,32^ According to Fernández-García et al. and del Río et al., Au^δ−^ are the most active sites,^33,34^ but CO oxidation also occurs at less-active Au^δ+^ sites.^34^ Using in situ diffuse reflectance infrared Fourier transform spectroscopy (DRIFTS), del Río et al. showed that CO adsorbs on neutral and negatively charged Au sites. They introduced O_2_ into the DRIFTS cell to induce the CO oxidation reaction, leading to the rapid oxidation of CO species adsorbed on Au^δ−^. In contrast, Au^0^–CO species were converted to Au^δ+^–CO prior to a comparatively slower oxidation of CO to CO_2._^34^ Thus, the direct and unequivocal detection of Au^δ−^ sites as well as the determination of their formation conditions, is crucial to improve the activity of Au/ceria catalyst in CO oxidation on a knowledge-driven basis. A few theoretical studies predict the formation of Au^δ−^ sites as a result of charge transfer from oxygen vacancies to gold.^31,32,35^ A DRIFTS peak assigned to CO–Au^δ−^ was also observed on Au/oxide systems by Fernández-García et al.^33^ Although infrared (IR) spectroscopy using CO as a probe molecule is well-suited to study the nature and abundance of this adsorbate under realistic conditions, it only provides an indirect indication of the electronic state of the underlying oxide substrate.^36^

In principle, binding energy (BE) shifts revealed by X-ray photoelectron spectroscopy (XPS) could provide direct information about the electronic states of Au atoms. However, the experimental investigation of the charge state of the active Au sites in Au/ceria catalysts at realistic CO oxidation conditions is challenging. Foremost, the charging effects on nonconductive powder ceria samples impede the accurate fitting of Au 4f spectra and, consequently, make it essentially impossible to distinguish the signal of Au^δ−^ unequivocally. Some of us observed a strong broadening of Au 4f spectra of Au/CeO_2_ NPs at temperatures of <150 °C,^37^ which is the typical temperature of CO oxidation on Au/CeO_2_ catalysts.^1,38,39^ Charging effects are practically absent in measurements of the “model” samples, where a CeO_2_ layer a few nanometers thick is grown on conductive Cu(111), Ru(0001), or Si(100) supports.^40−42^ Matolín et al. showed well-defined XPS signals related to Au^0^, Au^+^, and Au^3+^ in a magnetron-sputtered Au/CeO_2_/Si(100) sample.^43^ Despite the abundance of studies addressing the formation of Au^δ−^ sites on ceria-supported Au samples, none reports the detection of such Au^δ-^ species employing XPS.

To address this challenge, we studied powder (real) and thin film-based (model) catalyst samples using different experimental techniques, drawing analogies between the two types of samples and focusing on the differences between resting and operando regimes of the catalysts. First-principles modeling based on density functional theory (DFT) allows us to further characterize these systems and their behavior in atomic detail.

We start by describing the structure of the ceria-supported Au particles in the absence of reactants by employing high-angle annular dark-field scanning transmission electron microscopy (HAADF-STEM) on powder samples. The morphology and electronic structure of analogous ceria-supported Au particles in the well-defined model catalysts are then evaluated by scanning tunneling microscopy (STM) and XPS, including high-resolution measurements using synchrotron radiation (SRPES). This model approach minimizes the charging effects described above. We rationalize the XPS results with the aid of calculated electronic charge distribution and core-level binding energy (BE) shifts.

We continue by describing the properties of Au/CeO_2_ exposed to CO. In particular, we perform near ambient pressure (NAP)-XPS characterization of the model Au/CeO_2_ system to explore how the electronic structure evolves under reaction with CO. We relate this to calculated changes in the electronic structure and core-level BEs induced by the reduction of the Au/CeO_2_ model to pinpoint the origin and properties of Au^δ−^ species. In order to relate the changes in the electronic structure to particular chemical properties of the catalysts and elucidate the nature of CO adsorption sites, we perform DRIFTS measurements for the powder Au/CeO_2_ sample using CO both as a reactant and as a probe molecule. The DRIFTS studies of the as-prepared powder Au/CeO_2_ catalyst were performed at temperatures between 25 °C and 150 °C. To aid the assignment of the CO peaks, we compared the experimental peak positions to vibrational frequencies of CO adsorbed on the computational Au/CeO_2_(111) model calculated by DFT calculations.

## 2 Methods

### 2.1 Sample Preparation and Characterization

The powder sample—ceria nanooctahedra decorated with Au NPs—was synthesized by the wet chemistry method described in detail previously.^44,45^ Briefly, the support was synthesized by a microwave-assisted hydrothermal method. 0.4370 g of cerium nitrate Ce(NO_3_)_3_**·**6H_2_O was first dissolved in 35 mL of distilled water. Next, the obtained solution was mixed with 5 mL aqueous solution of 0.005 g of sodium phosphate (Na_3_PO_4_) and then stirred for 60 min. The resulting solution was treated at 170 °C for 1 h under autogenous pressure in an autoclave to obtain octahedral nanocrystals. The as-obtained precipitate powder of ceria nanooctahedra was washed and dried at 60 °C for 12 h. 200 mg of the washed and dried ceria nanooctahedra, 8 mg of H[AuCl_4_], 512 mg of (NH_2_)_2_CO, and 12 mL of H_2_O were mixed to form a suspension. The suspension was stirred and kept at 80 °C in a silicone oil bath for 24 h. Au/ceria particles were washed, dried at 50 °C for 12 h, and annealed in air (or in H_2_) at 300 °C for 3 h.

**Figure 1:**
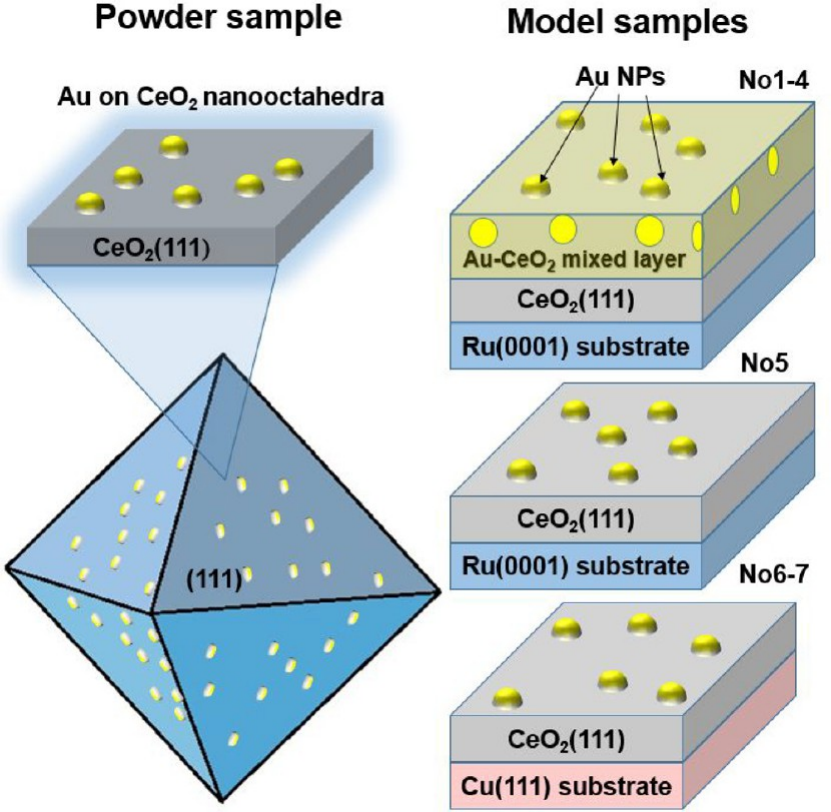
Scheme of the powder Au/CeO_2_ and model samples: Au–CeO_2_/CeO_2_/Ru(0001) (samples 1–4), Au/CeO_2_/(Ru(0001) (sample 5) and Au/CeO_2_/Cu(111) (samples 6 and 7).

Two different types of model samples were prepared. The samples of the first type contain Au homogeneously dispersed in stoichiometric and reduced ceria (Au–CeO_2_/CeO_2_ and Au–CeO_2–*x*_/CeO_2_ (*x* < 0.5), Nos. 1–4), while the others contain Au in the form of deposited particles on the surfaces of stoichiometric and partially reduced ceria (Au/CeO_2_ and Au/CeO_2–*x*_, Nos. 5–7). Samples 1–7 are schematically depicted in Figure 1 and are described in detail in Table 1. They resemble the powder catalyst samples mentioned above, where Au NPs are deposited on ceria nanooctahedra exposing {111} facets.

**Table 1:** Summary of the Structural Properties and Preparation Conditions of the Model Catalysts Samples

No.	sample composition	deposited material	*T*_Au dep_ [°C]	*d*(CeO_2_) [nm]	*d*(Au–CeO_2_) [nm]	Au–CeO_2_ structure
1	Au–CeO_2_/CeO_2_/Ru(0001)	Au–CeO_2_	300	1.5	2.5	(111)
2	Au–CeO_2_/CeO_2_/Ru(0001)	Au–CeO_2_	25	1.5	3.5	amorphous
3	Au–CeO_2_/CeO_2_/Ru(0001)	Au–CeO_2_	–150	2.0	3.5	amorphous
4	Au–CeO_2–*x*_/CeO_2_/Ru(0001)	Au–CeO_2–*x*_	–150	2.0	3.5	amorphous
5	Au/CeO_2_/Ru(0001)	Au	25	2.0	–	–
6	Au/CeO_2_/Cu(111)	Au	25	2.0	–	–
7	Au/CeO_2–*x*_/Cu(111)	Au	25	2.0	–	–

The model samples were prepared by physical vapor deposition (PVD) in an ultrahigh vacuum (UHV) chamber with base pressures below 5 × 10^–9^ mbar. Undoped CeO_2_ and CeO_2-x_ layers 1.5–2 nm thick were deposited by evaporating Ce in an oxygen atmosphere (*p*_O_2__ = 5 × 10^–7^ mbar and 1 × 10^–8^ mbar, respectively) on Ru(0001) or Cu(111) substrates at 250 °C. Both single-crystal metal supports are suitable for epitaxial growth of the oriented CeO_2_(111) thin film.^41,46,47^ Low-energy electron diffraction (LEED) patterns obtained from the CeO_2_(111) layers prepared on the Cu(111) and Ru(0001) were identical (Figure S1 of the Electronic Supporting Information (ESI)). They confirm the CeO_2_ structure and (111) surface orientation. To prepare the Au/CeO_2_ and Au/CeO_2–*x*_ system (sample Nos. 5–7), 0.2 monolayers (ML) Au were deposited on the stoichiometric and partially reduced cerium oxide surfaces by Au evaporation in UHV at 25 °C. The mixed Au–CeO_2_ or Au–CeO_2–*x*_ layers were prepared by simultaneous Ce and Au metal deposition on the top of a CeO_2_ buffer layer in *p*_O_2__ = 5 × 10^–7^ mbar or 1 × 10^–8^ mbar, respectively. The epitaxial CeO_2_(111) buffer layer was used to prevent possible Au–Ru or Au–Cu interactions. The total thickness of the Au–CeO_2_/CeO_2_ and Au–CeO_2-x_/CeO_2_ layers (including the buffer layer) ranged from 2.5 nm to 3.5 nm (cf. Table 1), while the atomic concentration of Au was ∼10% in all cases. These samples differ in the resulting stoichiometry and morphology of the formed composite due to the different oxygen pressure and temperature during the film growth. Specifically, codeposition temperatures were 300 and 25 °C for samples 1 and 2, respectively, and −150 °C for samples 3 and 4. The preparation and study of the composite Au–CeO_2_ and Au–CeO_2–*x*_ layers were justified by creating a maximal contact area between Au and ceria (Au|CeO_2_ or Au|CeO_2–*x*_), which provided almost ideal conditions for the investigation of MSI between Ce NPs and Au NPs. The deposition temperature was varied to prepare nanostructured model ceria layers containing gold with different dispersions. Subnanometer clusters or even a single-atomic gold dispersion were expected for temperatures of −150 °C due to the limited surface diffusion of Au atoms. The surface morphology of the ceria layer is also temperature-dependent, and a ceria surface with a higher amount of atomic steps (leading to higher Au dispersion) is usually prepared by decreasing the deposition temperature. In turn, the growth of nanometer-size particles takes place at higher temperatures.^48^

All model samples were prepared in the preparation chambers of the NAP-XPS and SRPES facilities. For a more-detailed description of the preparation procedure, see the ESI.

HAADF-STEM images of the powder sample were obtained with a Cs probe-corrected FEI TITAN microscope operating at 300 kV. Specimens were prepared by dispersing the sample in methanol and putting a droplet of the suspension on a microscope copper grid covered with carbon. Samples were then dried and purified in oxygen/argon plasma in a plasma cleaner.

The morphology of model sample 5, best resembling the powder sample structure, was examined by scanning tunneling microscopy (STM). The STM measurements were performed using a high-resolution SPM Aarhus 150 microscope in a separate UHV chamber connected to the preparation chamber of the NAP-XPS system. The sample was transferred there from the preparation chamber without breaking the vacuum. The pressure in the STM chamber was <5 × 10^–10^ mbar. The microscope was operated in the constant current mode by using a Specs Kolibri Sensor. The scanning process was controlled by a Nanonis control system.

### 2.2 DRIFTS Measurements

The DRIFTS measurements of the powder sample were performed with a Vertex 80v spectrometer (Bruker) equipped with a KBr beam splitter and a liquid-N_2_-cooled HgCdTe detector. The sample compartment of the spectrometer is enclosed in a home-built housing, which features a reactor chamber and a diffuse reflectance accessory (both from Harrick). The reactor chamber is equipped with KBr windows and a type K thermocouple. The complete optical path is evacuated during the measurements to achieve stable measurement conditions.

In the DRIFTS experiments presented herein, we used Ar (Linde, >99.999%), O_2_ (Linde, >99.999%), and CO (Linde, >99.997%). CO was passed through two carbonyl traps (Gaskleen II Purifier from Pall Corporation and a heatable carbonyl trap from Leiden Probe Microscopy) to remove metal carbonyls. Gas flow and pressure in the setup are regulated between 0–20 mL_N_/min and from 1 mbar to 20 bar by mass flow and pressure controllers (both from Bronkhorst). The complete setup is remote-controlled to allow long-term experiments with precise control of all experimental parameters (gas flow, gas composition, pressure, temperature).

The experimental procedure was identical to that used in our previous work.^37^ Briefly, the samples were purged overnight with an Ar stream to remove water traces. To remove contaminations, the samples were heated in 20 vol % O_2_ in Ar (total pressure 1 bar) at 300 °C. After cooling to room temperature, a reference spectrum was recorded in 1 bar Ar (acquisition time 2 min, spectral resolution 2 cm^–1^). Subsequently, spectra were recorded continuously during the experiment (acquisition time 1 min). For each temperature step (30, 50, 100, and 150 °C), the samples were first exposed to 5 vol.% CO in Ar (total pressure 1 bar, 14 min), during which CO adsorbs on Au. This is followed by a stepwise evacuation (24 min, ∼1 mbar) to remove the CO gas phase. Finally, the samples were treated oxidatively in 20 vol % O_2_ in Ar (total pressure 1 bar, 18 min) to remove the remaining adsorbed CO and reoxidize the support. The procedure was applied throughout a heating/cooling process, heating in a stepwise manner at 30, 50, 100, and 150 °C and later cooling to 100, 50, and 30 °C. Post-data treatment of the spectra includes normalization to account for changes in the reflectivity upon heating.^49^ Finally, the gas-phase CO signal in the spectra was removed numerically,^37,50,51^ and the spectra were baseline-corrected.

### 2.3 NAP-XPS Measurements

NAP-XPS measurements of the model samples were performed on the NAP-XPS system (Specs).^37^ The regions of interest during the NAP-XPS measurements were Ce 3d, Au 4f, O 1s, C 1s, and Ru 3d. They were recorded at pressures ranging from 1 × 10^–9^ (UHV) up to 1.4 mbar and temperatures ranging from −150 °C to 300 °C, using a monochromatized Al Kα (1487 eV) X-ray source of high intensity.

High-resolution SRPES measurements were performed at the Materials Science Beamline (MSB), Elettra Synchrotron Light Facility in Trieste, Italy.^52,53^ The Ce 3d, Au 4f, O 1s, C 1s, and Cu 2p regions were recorded in UHV at room temperature using an Al Kα X-ray source and also Au 4f region using synchrotron radiation (180 eV).

The nominal thicknesses of the deposited CeO_2_(111), CeO_2–*x*_(111), Au–CeO_2,_ Au–CeO_2–*x*_, and Au layers were calculated from the attenuation of Ru 3d_5/2_ or Cu 2p_3/2_ peak intensities, using the equation
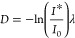
where *I*_0_ is the initial peak intensity, I^*^ the peak intensity after the CeO_2_, Au–CeO_2_ or Au layer deposition, and λ the electron inelastic mean free path in CeO_2_ (19.68 and 11.19 Å for Ru 3d_5/2_ and Cu 2p_3/2_ photoelectrons, respectively). The NAP-XPS and SRPES spectra have been processed in the KolXPD software.

In order to rule out that changes in the oxidation state of the ceria substrate were induced by the X-ray beam (as reported by Paparazzo et al.),^54^ our measuring procedure for all XPS spectra included the collection of the Ce 3d region at the beginning and at the end of each measurement and no variations were observed.

### 2.4 Computational Details

The DFT calculations were performed using the VASP code^55,56^ and the generalized-gradient PW91 exchange-correlation functional.^57^ In order to partially correct the self-interaction error characteristic of such semilocal functionals, we used a GGA+U approach by introducing an on-site Coulombic interaction term *U* to Ce 4f orbitals. This approach satisfactorily describes the localized character of the electrons occupying Ce 4f orbitals in reduced ceria (despite disregarding long-range dispersion interactions). Following previous work on similar ceria-supported metal particles, we used a *U* value of 4 eV, leading to the PW91 + 4 approach.^58−62^

The structural model used to represent Au NPs supported on CeO_2_(111) contains a Au_31_ particle cut from the fcc crystal structure of bulk Au. The Au particle is terminated by small {111}-like facets, with the facet at the interface aligned epitaxially to the CeO_2_(111) surface to maximize the number of Au–O bonds formed and to match epitaxial alignments identified in this work and elsewhere.^21,22^

The Au_31_ particle has a diameter of ∼1 nm and is thus smaller than the ones found in the experimental samples (vide infra). Therefore, the Au_31_ particle model does not contain all features of the larger particles, but represents their low-coordinated sites and the Au/CeO_2_ interface quite well. Since it consists mostly of interfacial and undercoordinated atoms, the crystal structure is more distorted than more bulklike regions of larger particles. However, considering size-representative particles at the DFT level of theory is computationally unfeasible, and the Au_31_ model allows us to evaluate the charge distribution at the interface, the effects of Au undercoordination, and the electronic structure of the system in the presence of oxygen vacancies in partially reduced CeO_2-x_. However, extrapolations of the obtained results to larger particles must be performed with care. The CeO_2_(111) surface was represented by a periodic slab model, consisting of a 3 × 3 supercell with 9 atomic layers (three O–Ce–O trilayers), and using the experimental lattice parameter of CeO_2_ (∼5.40 Å). Since the reduction of Ce^4+^ cation to Ce^3+^ increases the volume of the Ce atom, we use the experimental lattice parameter to avoid any volume-related biases to calculated properties involving ceria reduction. The electronic structure of this model was calculated self-consistently with an energy convergence threshold of 10^–6^ eV. Valence electrons were described using a plane-wave basis set with a kinetic energy cutoff of 415 eV. The interaction between core and valence electrons was taken into account using the projector-augmented wave method of Blöchl.^63^ During geometry optimization, the bottom three atomic layers (one O–Ce–O trilayer) were kept fixed, whereas the rest of the atoms were allowed to relax locally until maximum atomic forces acting on them were lower than 0.05 eV/Å. We note that reducing the convergence criteria from 0.05 to 0.02 eV/Å results in differences in calculated CO adsorption energy values below 20 meV.

**Figure 2:**
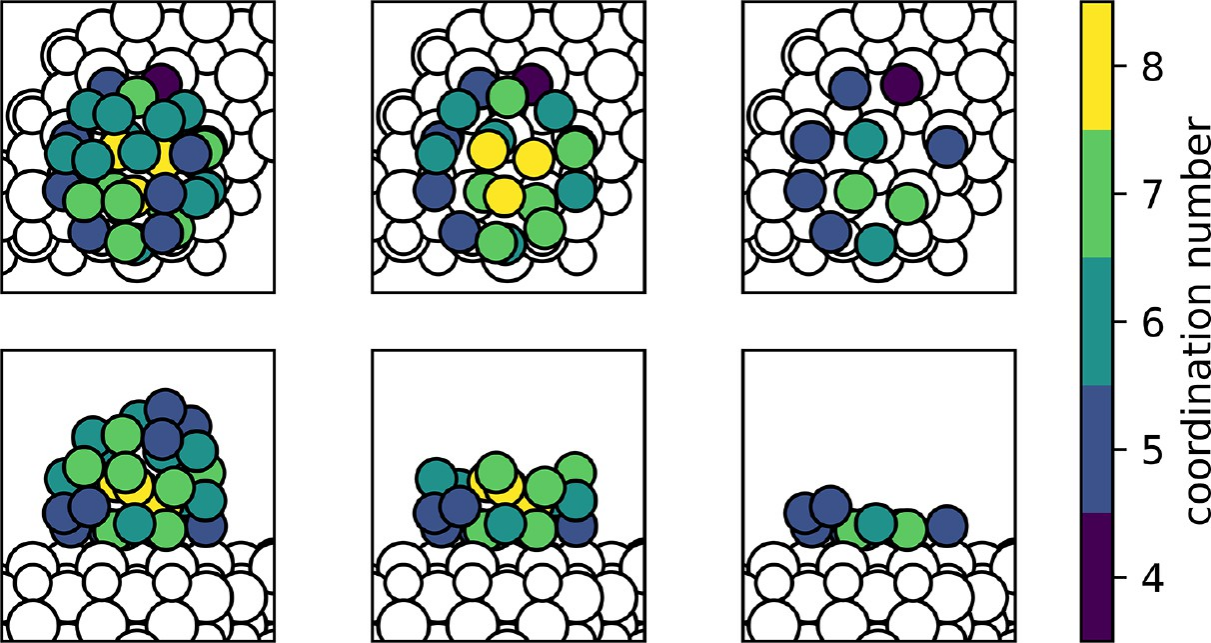
Top and side views of the optimized Au_31_/CeO_2_(111) model used to represent the ceria-supported Au NPs. Au atoms are color-coded according to their coordination number, and Ce and O atoms are shown as small and large white spheres, respectively. Some Au atoms in the images in the middle and right columns have been made transparent for clarity.

The resulting optimized Au_31_/CeO_2_(111) model is depicted in Figure 2, where Au atoms are color-coded according to their coordination number. For the relaxed structure, the average length of the Au–O and Au–Au bonds is 2.21 and 2.83 Å, respectively, with very similar Au–Au distances between interface and noninterface Au atoms. To obtain different electronic states of the Au_31_/CeO_2_ system, we allowed the VASP code to converge to different solutions by imposing different spin states (i.e., the difference between up and down electrons) upon an initial relaxation. The resulting structures/states were subsequently reoptimized starting from their converged wave function and geometry but lifting the spin multiplet restriction.

To rationalize DRIFTS data, harmonic vibrational frequencies of CO molecules adsorbed on selected Au atoms of the Au_31_/CeO_2_(111) model were calculated. Sites from different layers and with different coordination numbers were considered. For the calculation of the vibrational frequencies, the degrees of freedom of only the CO molecules were considered. As done in previous work,^64^ to account for the known underestimation of semilocal exchange-correlation functionals of the vibrational frequencies of CO, calculated frequencies were shifted by the difference between the calculated (2131 cm^–1^) and experimental (2143 cm^–1^) stretching frequency of the gas-phase CO molecule, i.e., ν = ν_calc_ – ν_calc_(CO-gas) + ν_exp_(CO-gas).

To interpret experimental XPS data, calculations of core-level BEs were performed relying on the Slater–Janak transition-state approximation.^65−68^ In this approach, the BE of a core electron (the energy difference between neutral *E*(*N*) and electron-deficient core-hole *E*(*N* – 1) states) is computed via the Taylor series expansion up to the second term in the change of the occupation number of the core orbital under scrutiny. This binding energy equals the eigenvalue of the corresponding half-occupied core orbital:
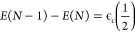
where *N* is the total number of electrons of the system and  is the eigenvalue of the half-occupied core Kohn–Sham state. Note that, in the implementation of this approach in the VASP code, the screening by other core electrons is not taken into account, i.e., core electrons are kept frozen. Therefore, the calculation of  values only accounts for the screening of the core–hole by valence electrons. Hence, this approach cannot be used to calculate absolute values of the core electron binding energies, although it is reported to provide core electron BE shifts with an accuracy of better than 50 meV.^69^

## 3 Results and Discussion

The structure and properties of the Au/ceria catalysts at rest and under CO exposing conditions were investigated through multiple experimental and theoretical techniques. The experiments involved two groups of Au/CeO_2_(111) samples: powder samples consisting of Au NPs deposited on ceria nanooctahedra (mainly terminated by {111} facets) and model samples where Au NPs were either deposited on or dispersed in a thick film of (111)-terminated or amorphous ceria that is a few nanometers thick. We focus on the structure and oxidation states of Au (Au^δ+^, Au^0^, and Au^δ−^) after their preparation and evolution under interaction with CO molecules. Although the model and powder samples are, in principle, different, they exhibit a similar interface between the Au particles and CeO_2_(111), which allows us to draw analogies between the two systems. We, therefore, assume that the effects resulting from the MSI and exposure to CO will be similar in both cases. Also, we note that this work is not focused on the investigation of catalytic activity of the prepared samples toward CO oxidation, but rather on the structural and electronic interplay between gold and ceria in the Au/CeO_2_(111) catalyst before and after exposure to CO. This information might help elucidate the reaction mechanism of low-temperature CO oxidation on Au/CeO_2_ catalysts, which is still under debate.^70^ The catalytic activity of the powder sample was studied in detail in our previous works,^44,71^ showing CO oxidation already at room temperature (an example of the light-off plot for it is shown in Figure S2 in the ESI). An investigation of CO oxidation over Au NPs deposited on epitaxial CeO_2_(111) film grown on Ru(0001) is presented in ref (72) and demonstrates their high activity at room temperature.

### 3.1 Resting State of Au/CeO_2_ Catalyst

#### 3.1.1 Structure

**Figure 3:**
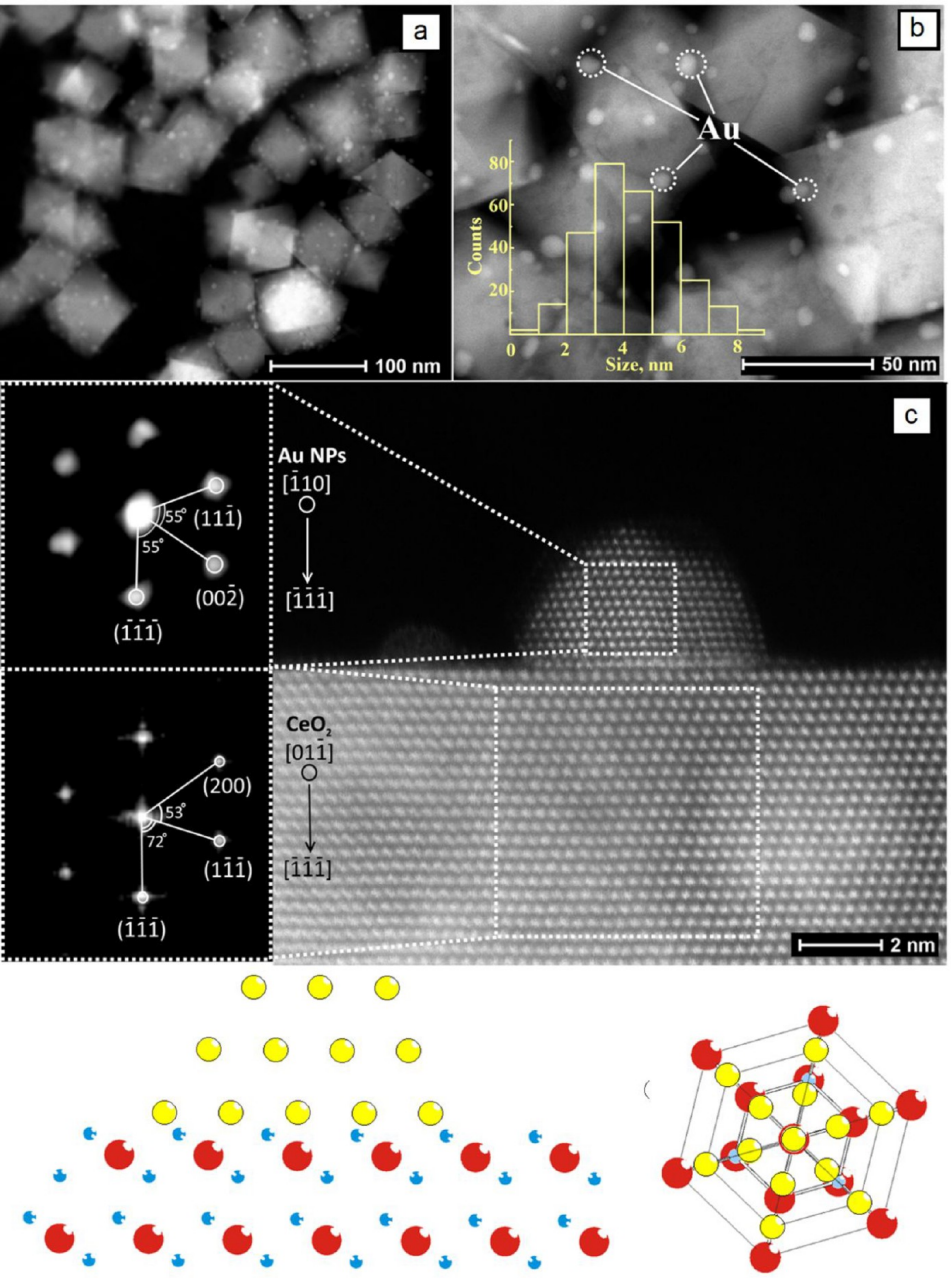
(a, b) Low-magnification HAADF images of Au/CeO_2_, with the size distribution of Au NPs plotted in the inset; (c) atomic resolution HAADF image of Au/CeO_2_, with Fast Fourier transform of selected areas on the left side. The bottom part of the figure schematically illustrates the mutual orientation of Au NPs and CeO_2_(111) support; color coding of atoms: Au, yellow; Ce, red; and O, blue.

HAADF-STEM images of the Au NPs supported on CeO_2_ nanooctahedra are shown in Figures 3a and 3b. The ceria support in the powder sample corresponds to crystalline nanooctahedra terminated by {111} facets.^44,73^ The gold content in the powder Au/CeO_2_(111) samples, estimated by energy-dispersive X-ray spectroscopy (EDX), was ∼1 at. %. The Au particle size distribution shows the typical Gaussian shape with a maximum at ∼4 nm.

High-resolution HAADF-STEM images of the Au/CeO_2_(111) catalyst revealed a preferential orientation of Au crystallites relative to the CeO_2_ lattice, with [111] Au parallel to the [111] plane of CeO_2_ (Figure 3). This observation agrees well with the data published by our group^45^ and other authors.^74,75^ Moreover, Au NPs are rotated relatively to the CeO_2_ support by ∼60° (cf. scheme in Figure 3c), so that the [1̅10] direction of Au NPs coincides with the [011̅] direction of CeO_2_. The structural model used for the DFT calculations presented in this work was created taking these HAADF-STEM data into account by placing the (111)-like termination of the Au_31_ particle on the CeO_2_(111) surface, forming a similar epitaxial orientation (see Figure 2).

**Figure 4:**
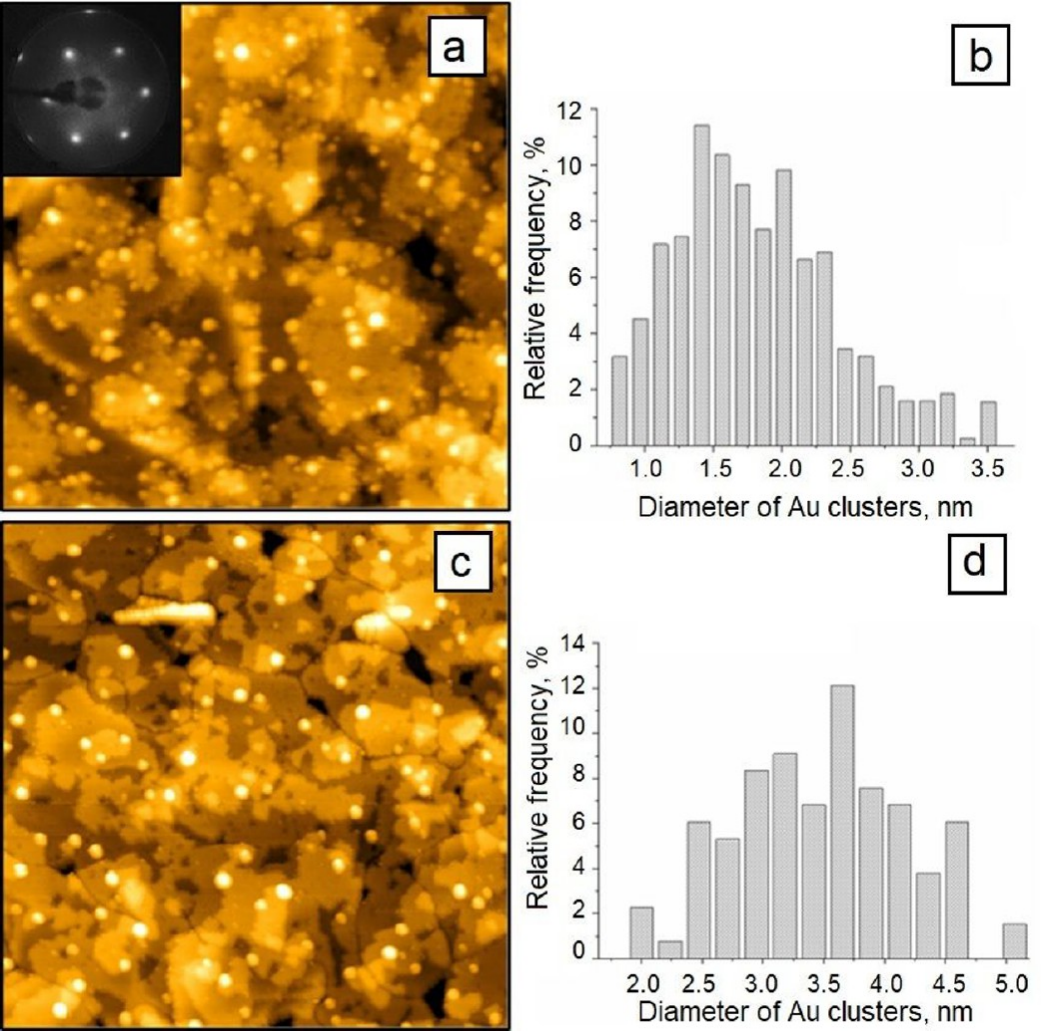
STM images (105 nm × 105 nm) of 0.2 ML Au deposited on CeO_2_(111) at 25 °C (a) and after annealing at 300 °C in UHV for 2 min (c) with the corresponding histograms of the particle diameter distribution (panels (b) and (d), respectively). The STM images were measured in the constant current mode using the current of 6 pA and voltage of +4 V.

A high-resolution STM image of the as-deposited Au nanoparticles on the model CeO_2_(111) surface (sample 5) is presented in Figure 4a. The inset image shows the LEED pattern of the ceria surface before the deposition of gold. It can be seen that the Au NPs decorate mainly ceria steps, which is in good agreement with the literature data.^76^ As can be seen from the histogram of Au NP diameter distribution (Figure 4b), the average size of the as-deposited Au NPs was ∼1.5 nm. We also characterized the sample after 2 min annealing at 300 °C (Figures 4c and 4d). The annealing resulted in the nanoparticle growth to sizes comparable with the size of gold particles on the powder sample (Figure 4d). A similar particle growth also occurs during the annealing of this sample in the presence of CO. Indeed, the high-pressure studies performed on such a model system^76^ demonstrated that Au particles, exposed to CO or a mixture of CO and O_2_ in the mbar range, start to coalesce already at room temperature. The increasing temperature should only accelerate this process.

#### 3.1.2 Oxidation States of Au and Ce

**Figure 5:**
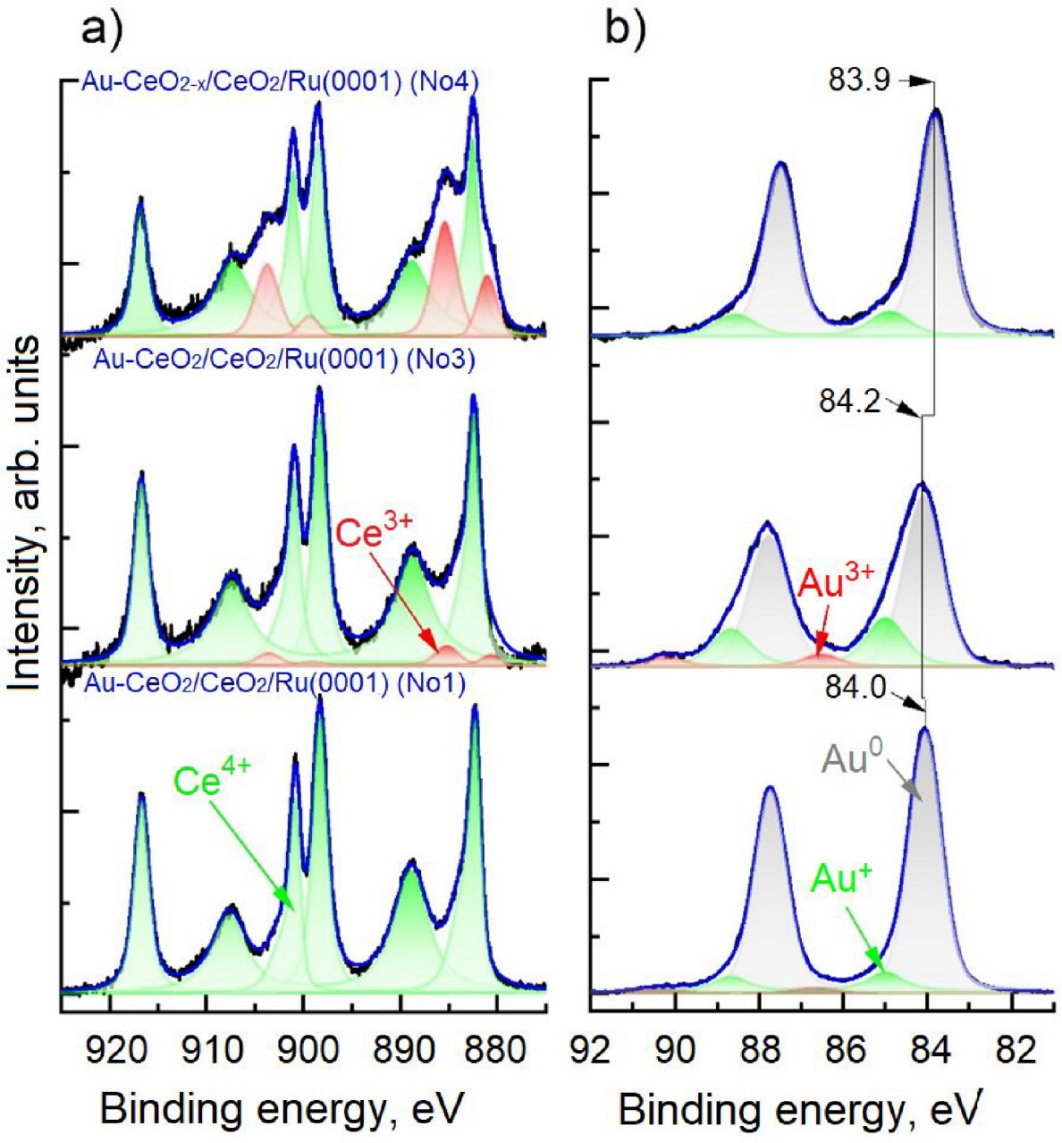
Normalized XPS Ce 3d (panel (a)) and Au 4f regions (panel (b)) of the UHV spectra acquired at 25 °C on the stoichiometric Au–CeO_2_/CeO_2_/Ru(0001) (sample 1, Au–CeO_2_ codeposited at 300 °C), stoichiometric Au–CeO_2_/CeO_2_/Ru(0001) (sample 3, Au–CeO_2_ codeposited at −150 °C), and nonstoichiometric Au–CeO_2–*x*_/CeO_2_/Ru(0001) (sample 4, Au–CeO_2–*x*_ codeposited at −150 °C). Color coding: recorded Ce 3d spectra (black), three Ce^4+^ related doublets (green), two Ce^3+^ related doublets (red), and a sum fit (blue). Measured Au 4f spectra (black), their fitting in Au^3+^ (red), Au^+^ (green), Au^0^ (gray), and a sum fit (blue).

**Figure 6:**
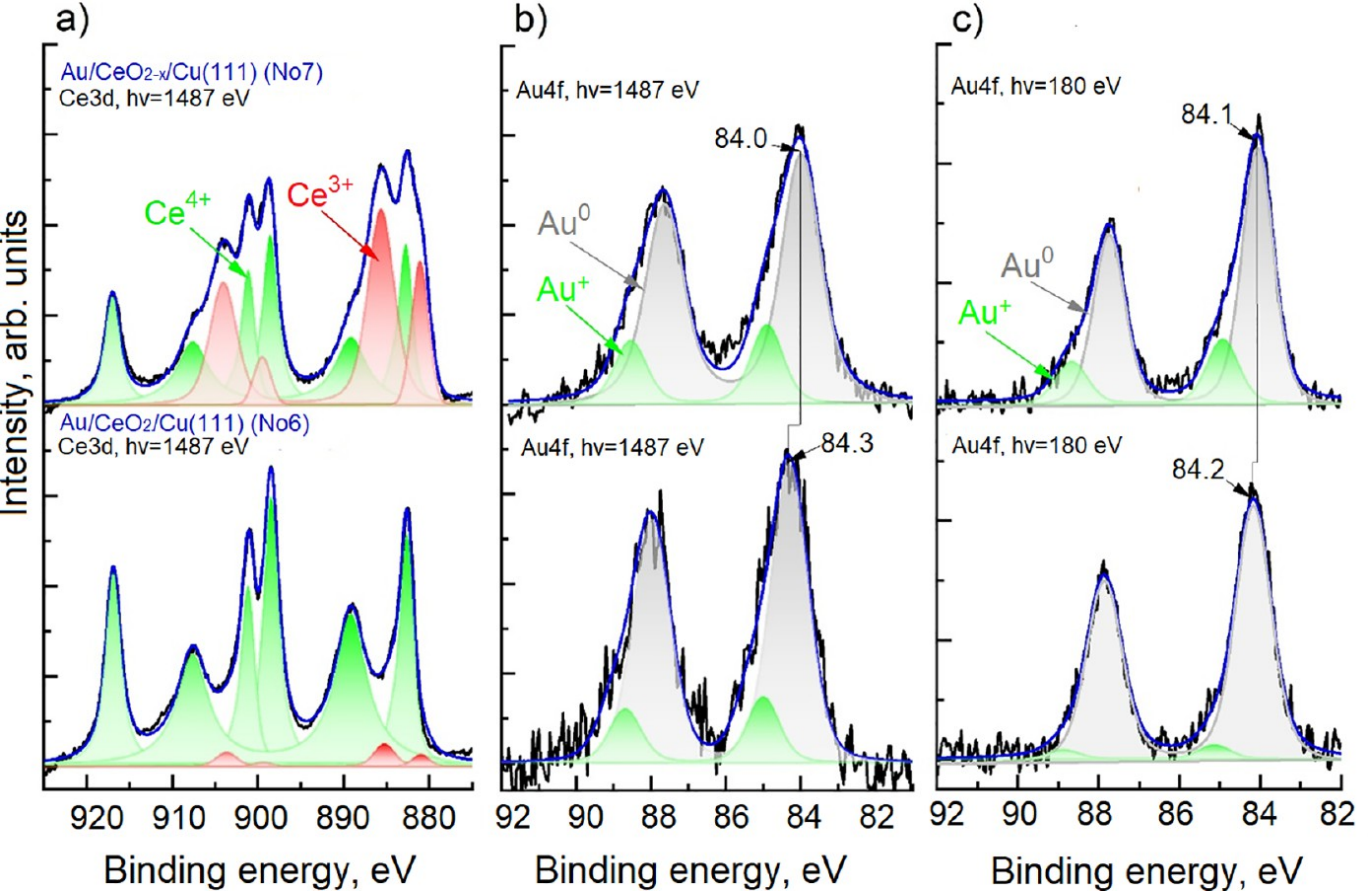
Normalized XPS Ce 3d (panel (a)) and Au 4f (panel (b)) and SRPES Au 4f (panel (c)) spectra of stoichiometric Au/CeO_2_/Cu(111) (sample 6) and nonstoichiometric Au/CeO_2–*x*_/Cu(111)) (sample 7) samples. Color-coding for Ce 3d regions: measured spectra (black), three Ce^4+^ related doublets (green), two Ce^3+^ related doublets (red) and a the sum of both components (blue). Color-coding for Au 4f regions: measured spectra (black), fitted components Au^+^ (green), Au^0^ (gray), and their sum (blue).

Next, we focus on the electronic structure of the ceria-supported Au particle as induced by the MSI. We studied the model samples using XPS in UHV. Ce 3d and Au 4f spectra were of primary interest for providing information about the gold–ceria interaction. The characteristic spectra of the mixed Au–CeO_2_ Au–CeO_2–*x*_ samples (Nos. 1, 3, and 4) are shown in Figure 5, while the spectra of the Au/CeO_2_ and Au/CeO_2–*x*_ surfaces (Nos. 6 and 7) are presented in Figure 6. The corresponding spectra measured on samples 2 and 5 are depicted in Figure S3 of the ESI. All spectra reveal the presence of various Au and Ce species. The oxidation state of Ce was evaluated by the fitting procedure of the Ce 3d spectra described elsewhere.^77^ The oxidation state of the Au NPs was determined by subtracting a Shirley-type background and subsequently deconvoluting the Au 4f spectra into three Voigt doublets (Figure 5). The first doublet at BE of ∼84/87.7 eV corresponds to metallic Au^0^, the second at ∼85/88.7 eV is associated with the oxidized Au^+^ species, and the third at ∼86.5/90.2 eV with oxidized Au^3+^ species embedded in the ceria lattice.^43^ Features of Au^δ−^ species would be expected to appear at lower BEs with respect to Au^0^, but no such peaks were detected in any of the spectra recorded. The relative contributions of the fitted components representing the different oxidation states of Ce and Au are summarized in Table 2.

**Table 2:** Relative Contribution of Oxidation States of Ce and Au for All Au–Ceria Model Samples Studied Using XPS and SRPES in Ultrahigh Vacuum (UHV)^a^

			Ce 3d		Au 4f				
No.	sample composition	measurement conditions	Ce^3+^ (%)	Ce^4+^ (%)	Au^0^ (%)	Au^+^ (%)	Au^3+^ (%)	Au^3+^ + Au^+^ (%)	Au 4f_7/2_ peak position (eV)
1	Au–CeO_2_/ CeO_2_/Ru(0001)	UHV, 25 °C	<1	99	87	11	2	13	84.0
2	Au–CeO_2_/ CeO_2_/Ru(0001)	UHV, 25 °C	2	98	77	21	2	23	84.1
3	Au–CeO_2_/ CeO_2_/Ru(0001)	UHV, –150 °C	4	96	71	23	6	29	84.2
		UHV, 25 °C	4	96	86	10	4	14	84.1
4	Au–CeO_2–*x*_/ CeO_2_/Ru(0001)	UHV, –150 °C	19	81	73	20	<1	20	83.9
		UHV, 25 °C	23	77	77	13	<1	13	83.9
5	Au/CeO_2_/Ru(0001)	UHV, 25 °C	4	96	77	23	<1	23	84.3
6	Au/CeO_2_/Cu(111)	UHV, 25 °C	*h*ν = 1487 eV						
			4	96	83	17	0		84.3
					*h*ν = 180 eV (SRPES)				
					96	4	0	4	84.2
7	Au/CeO_2–*x*_/Cu(111)	UHV, 25 °C	*h*ν = 1487 eV						
			43	57	80	20	0	20	84.0
					*h*ν = 180 eV (SRPES)				
					80	20	0	20	84.1

a Relative contributions of the fitted components are calculated from the corresponding peak areas.

The UHV-XPS data (cf. Table 2) shows the presence of Au^0^ and Au^+^ species in all samples. There is also a tiny amount of Au^3+^ species in samples with the mixed stoichiometric Au–CeO_2_ layer (samples 1–3), which increases when decreasing the codeposition temperature. In contrast, there are no Au^3+^ species in the reduced Au–CeO_2–*x*_ sample (sample 4, Figure 5) and samples of Au NPs deposited on CeO_2_(111) (samples 5–7, Figure 6). This suggests that Au^3+^ species are incorporated in the ceria lattice during codeposition of Au and Ce in the samples with a mixed Au–CeO_2_ layer. These two forms of cationic gold (Au^+^ and Au^3+^) are frequently reported in XPS investigations of oxide–Au systems, including Au/CeO_2_.^78−80^ Literature data shows that Au^3+^ forms in two ways: upon exposure of gold to ozone as Au_2_O_3_ (unstable under ambient conditions),^81^ and in the mixed Au–ceria systems.^43,80^ In particular, using depth-resolved XPS data of radio-frequency (rf)-sputtered Au/CeO_2_ thin films, Matolín et al. demonstrated that Au^+^ species occur at the interface between Au NPs and the support, while Au^3+^ species were attributed to atomically dispersed Au in the bulk of CeO_2_.^43^ This explains why Au^3+^ is present in the codeposited Au-CeO_2_ mixed layers (samples 1–3) but not in the Au/CeO_2_ samples (samples 5–7). Also, it seems that the low oxygen pressure applied during the codeposition of Au and Ce (sample 4) is insufficient to form Au^3+^ ions in the layer.

Samples 6 and 7 were further investigated using the very surface-sensitive SRPES. Acquiring the spectra at different photon energies allowed us to evaluate the depth profile of the different Au oxidation states and to corroborate the assignment of Au^+^ species to interface Au atoms in contact with the ceria support. A comparison of SRPES (*h*ν = 180 eV) and UHV-XPS (*h*ν = 1487 eV) data obtained on sample 6 (Figure 6) reveal a significantly lower fraction of Au^+^ when using the lower photon energy, indicating that such species do not preferentially occupy surface positions of Au NPs. In the case of sample 7, the Au^+^ signal was high, even when measured with the low-energy photons (i.e., high surface sensitivity). This might be related to small gold clusters on the partially reduced surface of ceria. Indeed, it is known from the literature that oxygen vacancies at the surface of CeO_2_(111) are effective traps for Au atoms, which then become nucleation centers for particle growth.^82^ An increased number of nucleation centers, in turn, leads to the formation of smaller particles. In addition, the Au 4f spectra in Figure 6 also show that, irrespective of the photon energy used, there is always a 0.1–0.3 eV shift to lower BE in the position of the Au^0^ doublet for gold deposited on the partially reduced ceria surface. A similar shift also occurred for the Au–CeO_2–*x*_ sample (Figure 5), indicating that presence of oxygen vacancies in the vicinity of Au NPs influences their electronic structure independently regardless of the NPs being inside the layer or on its surface. As further discussed below, we assign this shift the charge transfer from ceria to Au NPs upon reduction of the oxide. However, we note that the charge of individual Au atoms after this transfer is dependent on their relative position within the NP and on their state before the reduction of the substrate.

**Figure 7:**
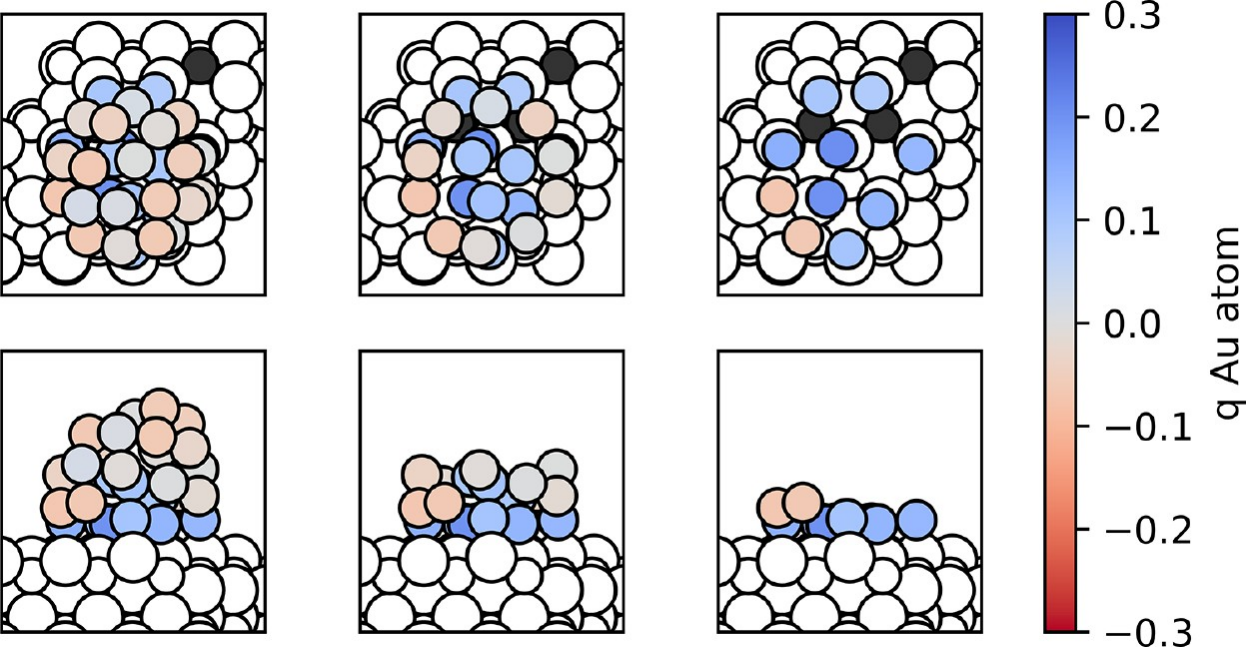
Top and side views of the most stable state found for the Au_31_/CeO_2_(111) model. Au atoms are colored according to their calculated Bader charge q. Ce^3+^ cations are displayed as black spheres and Ce^4+^ and O atoms as small and large white spheres, respectively. Some Au atoms in the images in the middle and right columns have been made transparent for clarity.

**Figure 8:**
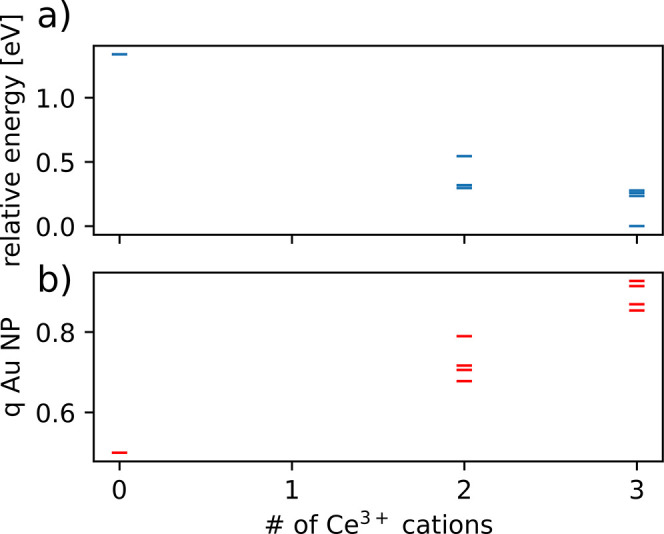
(a) Relative energies and (b) Au NP charge of different states calculated for the Au_31_/CeO_2_(111) model, as a function of the number of Ce^3+^ cations formed. Some Au atoms in the images in the middle and right columns have been made transparent for clarity.

The above XPS results are in good agreement with the charge distribution obtained in our DFT calculations of the Au_31_/CeO_2_(111) model (Figure 7). Bader atomic charges^83^ calculated for this system reveal that Au atoms at the interface with CeO_2_(111) are positively charged, while Au atoms at the surface positions of the NP are essentially neutral or slightly negatively charged. This charge distribution corresponds to the most stable state formed for this model, featuring three electrons transferred from the Au particle to the support, forming three reduced Ce^3+^ cations. We note that a sampling of different electronic states results in different numbers and positions of reduced Ce^3+^ centers (see Figure 8a). However, the overall Bader charge of the Au NPs remains positive even for states with no Ce 4f electrons formed (see Figure 8b).

Therefore, the situation for Au NPs is similar to that reported for ceria-supported Pt NPs,^59,60,84,85^ which are also partially oxidized upon interaction with the underlying oxide. In addition, the energy differences between the states of Au_31_/CeO_2_(111) with 2 and 3 Ce^3+^ cations are rather small, as are those between states with the same number of Ce^3+^ cations but occupying different positions. Considering the high mobility of polarons in ceria, the presence of quasi-degenerate states suggests a dynamic behavior of this system where the reversible and frequent transfer of electrons between the Au NP and different Ce cations could occur, similar to the behavior reported for Pt atoms supported on CeO_2_(100).^86^ This would involve that, in spectroscopic measurements, the average contributions of various configurations are evaluated instead of the single most stable one.

The assignment of the Au^+^ species to interface Au atoms allows us to rationalize the differences in relative fractions of Au^+^ on the basis of changes in the size or wetting properties of Au NPs. According to our previous work, the average size of deposited Au NPs in the Au/CeO_2_ system strongly increases with temperature.^71^ Thus, we assume that the average size (D) of the Au NPs in the Au–CeO_2_/CeO_2_/Ru(0001) samples follows a similar trend, with *D*(Au_300 °C_ – sample 1) > *D*(Au_25 °C_ – sample 2) > D(Au_–150 °C_ – samples 3 and 4). The surface-to-volume atomic ratio of Au NPs increases with decreasing particle size. Thus, the Au|CeO_2_ contact surface area decreases due to the formation of larger particles at elevated sample temperature during Au deposition. It is fully consistent with the differences in the measured relative concentration of oxidized Au species (both Au^+^ and Au^3+^) in samples 1–4 (see Table 2). More specifically, the fraction of the oxidized Au species varies from ∼13% to ∼29% when decreasing the co-deposition temperature from 300 °C (sample 1) to −150 °C (sample 4).

#### 3.1.3 Effect of Ceria Partial Reduction

So far, we have discussed the electronic structure of almost stoichiometric samples exhibiting low Ce^3+^ concentrations. Focusing on the differences observed for the understoichiometric (partially reduced) model samples provides information about the elusive Au^δ−^ species. Indeed, negatively charged gold species were reported to form because of a charge transfer from oxygen vacancies to Au atoms at the Au/CeO_2_ interface.^31,32^ There are also studies identifying Au^δ−^ sites by DRIFTS using CO as probe molecule,^33,37,87^ but none univocally detecting Au^δ−^ by XPS. Liu et al. reported Au^δ−^ XPS signal for Au/TiO_2–*x*_/ZnO system as shifted toward lower BE by 0.4 eV, with respect to Au^0^.^88^ However, XPS spectra presented in that work were recorded on nonconductive powder samples. This creates favorable conditions for strong inhomogeneous charging, resulting in peak broadening and problems with fitting. In the present study, such undesirable charging effects were avoided by using highly conductive Ru(0001) or Cu(111) single crystals as substrates for model samples.

As shown above, for both stoichiometric and nonstoichiometric samples, Au 4f regions are well-fitted by Au^0^, Au^+^, and Au^3+^-related doublets alone (Figures 5 and 6). We also found that the partial reduction of the ceria support resulted in a 0.1–0.3 eV shift of the entire Au 4f doublet toward lower BEs (Figures 5 and 6, and Table 2). In particular, Au 4f spectra acquired for the nonstoichiometric Au/CeO_2–*x*_ (sample 7) are shifted by 0.3 eV toward lower energy compared to the stoichiometric Au/CeO_2_ (sample 6, Figure 6). A similar shift of 0.2–0.3 eV was observed between reduced Au–CeO_2–*x*_/CeO_2_/Ru(0001) (sample 4) and its stoichiometric counterpart Au–CeO_2_/CeO_2_/Ru(0001) (sample 3). This suggests that some charge is transferred from the ceria support to the metal NPs when the ceria support is partially reduced.

To characterize the effect of ceria partial reduction on the electronic structure of the supported Au NPs, we investigated the formation of oxygen vacancies in the Au_31_/CeO_2_(111) model and calculated the Au 4f core-level BE shifts of all Au atoms in both reduced and nonreduced systems by means of the Slater–Janak transition state approach (see section 2.4, “Computational Details”).

The reduced Au_31_/CeO_2–*x*_(111) systems were formed by removing a single O atom from five different positions of the Au_31_/CeO_2_(111) model, including surface and subsurface O atoms both directly underneath and far from the Au NP. The structure of the resulting systems was locally relaxed to a (local) minimum of the potential energy surface. Relaxation of the reduced systems leads to a slight reconstruction of the Au particle, with larger horizontal distortions of Au–Au distances of the interface Au layer (with differences in average interatomic distances ranging from approximately −0.05 Å to approximately +0.05 Å) than for the rest of the Au particle. This is in agreement with the larger strain at interface positions identified by López-Haro et al. on Au/CeO_2_ by means of aberration-corrected high-resolution electron microscopy.^21^

**Figure 9:**
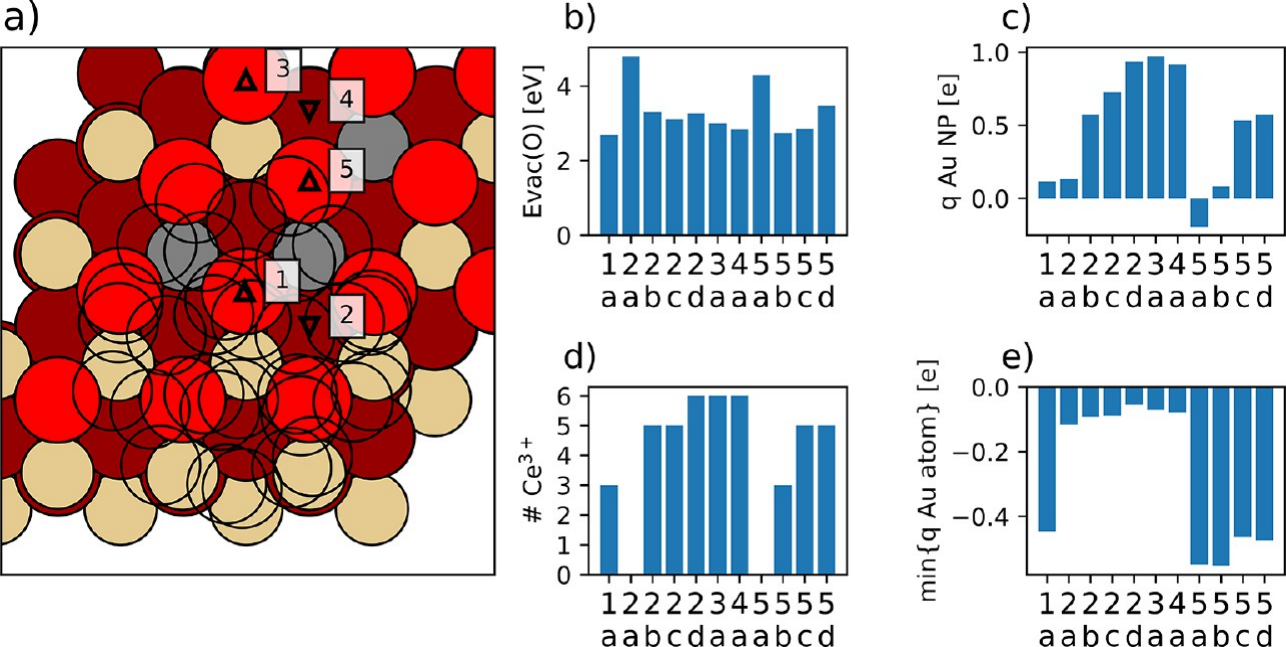
Calculated properties related to O vacancies created in different positions of the Au_31_/CeO_2_(111) model. Triangles pointing up and down in panel (a) indicate surface and subsurface positions, respectively. Surface O atoms, subsurface O atoms, Ce^4+^ cations, and Ce^3+^ cations are represented by bright red, dark red, beige, and gray circles, respectively. Au atoms are represented by nonfilled circles for clarity. Also shown are (b) O vacancy formation energies, (c) total charge of the Au NP, (d) number of Ce^3+^ cations formed, and (e) charge of most negatively charged Au atom in each structure, plotted for the different O vacancy positions (indicated by the corresponding numbers; see panel (a)) and different electronic states (indicated by letters).

As for the fully oxidized Au_31_/CeO_2_ system, the Au_31_/CeO_2–*x*_(111) model can feature several electronic states differing in the number and position of the Ce^3+^ cations. The two electrons left behind upon removing a neutral O atom can reduce additional Ce^4+^ cations to Ce^3+^ or may be transferred to the Au NPs. Therefore, the energy of the system is determined by both the position of the O vacancy and the distribution of Ce 4f electrons in the model. The O vacancy positions are illustrated in Figure 9a with triangles and corresponding numeric labels. O vacancy positions 2 and 5 are particularly interesting, because they correspond to the most stable O vacancy position in bare ceria and to a position in the vicinity of an Au atom exposed to reactants at the three-phase interface, respectively. Thus, more than one electronic state was explored for these vacancy sites. The states differ in the number and position of Ce^3+^ species, the total Ce 4f electron localization on each Ce^3+^ center, and the resulting Au NP charge and charge distribution (indicated with labels a–d). The resulting O vacancy formation energies (*E*_vac_(O), calculated with respect to half of the energy of a gas-phase O_2_ molecule in its triplet ground state) are plotted in Figure 9b. We also evaluated the number of Ce^3+^ cations (calculated as the number of Ce atoms with an absolute magnetic moment of >0.5 μ_B_) present after the formation of the vacancy (Figure 9c) and the resulting Bader charge of the Au NP (Figure 9d). Since the charge is not distributed homogeneously over the Au particle and to identify whether negatively charged Au^δ−^ species are formed, we also present the atomic charge of the most negatively charged atom (Figure 9e).

Forming O vacancies in the Au_31_/CeO_2_(111) system can lead to different numbers (from 0 to 6) of Ce^3+^ cations. However, we note that states with 5 or 6 Ce^3+^ centers often exhibit at least one delocalized Ce 4f electron (i.e., with magnetic moments from 0.5 to 0.7 μ_B_). This might give the impression that creating vacancies increases the number of Ce^3+^ centers by more than the expected maximum of two per O vacancy, but it just indicates that some f electrons are not fully localized. This also contributes to differences in the total Au NP charge between states with the same number of Ce^3+^ centers formed. States with no Ce^3+^ centers are notably unstable, with energies by ∼2 eV higher than the most stable state found. The energetically most stable state, with *E*_vac_(O) = 2.68 eV, corresponds to the O vacancy in position 1 (state a) and three formed Ce^3+^ cations. Interestingly, this state features a very negatively charged Au atom located right on top of the O vacancy. This is also the case for the next most stable state found with E_vac_(O)=2.74 eV, which also has three Ce^3+^ cations and an Au atom directly in contact with a surface O vacancy (position 5, state b) with a significant negative charge of approximately −0.6 **|e|** (as seen in Figure 9, as well as Figure S5 in the ESI). The charge distributions for the two states with negatively charged Au, displayed in Figures S4 and S5 in the ESI, show that a large fraction of the two electrons left behind by the removed O atom is preferentially transferred to an Au atom of the NP in contact with a surface O vacancy, whereas the other interface Au atoms remain in their positively charged (oxidized) states. These negatively charged species are similar to the single-atom Au^–^ sites found by Wang et al. by means of molecular dynamics simulations of Au supported on partially reduced ceria.^20^

Stable states without strongly negatively charged Au atoms were also found. For example, forming a vacancy in position 5 can also lead to an electronic state (5c) with 5 Ce^3+^ centers, a positively charged Au atom near the O vacancy, and *E*_vac_(O) = 2.86 eV. Similar to the stoichiometric system, the existence of states very close in energy with different number and distribution of Ce^3+^ cations suggests that the transfer of electrons between the Au particle and Ce atoms (and between different Ce atoms) could occur dynamically. Note that the deposited Au does not thermodynamically facilitate the removal of O atoms from the ceria surface, as revealed by the similarity between *E*_vac_(O) values for the most stable Au_31_/CeO_2-x_(111) states and for bare ceria (2.25–2.50 eV), calculated at the same level of theory.^60,89^

A more thorough sampling would likely find even more stable states, but the purpose of this study was to identify sufficiently representative states to characterize the general effects of the MSI in ceria-supported Au. In this regard, we conclude that interface atoms of Au NPs are slightly positively charged on stoichiometric CeO_2_(111), whereas the formation of O vacancies can lead to the presence of single negatively charged Au atoms in contact with the vacancy. Therefore, the total charge calculated for such Au particles remains positive and inhomogeneous.

We used the Slater–Janak transition-state approximation for calculating the BEs of Au 4f core–electrons of the different Au atoms in systems in different selected electronic states described above. In particular, we calculated the Au 4f BE for (a) the most stable state found for the Au_31_ particle supported on stoichiometric ceria, which corresponds to the charge distribution shown in Figure 7 (state 1a) with three electrons transferred from Au to Ce^4+^ cations; (b) an unstable electronic state for the Au particle supported on stoichiometric ceria with no electron transfer from the Au particle to Ce^4+^ (with ∼1.5 eV higher energy than the most stable state mentioned in panel (a); and (c) one of the most stable states found for the Au particle supported on the partially reduced ceria surface, where one Au atom near the O vacancy acquires a strong negative charge (see charge distribution in Figure S4 in the ESI).

**Figure 10:**
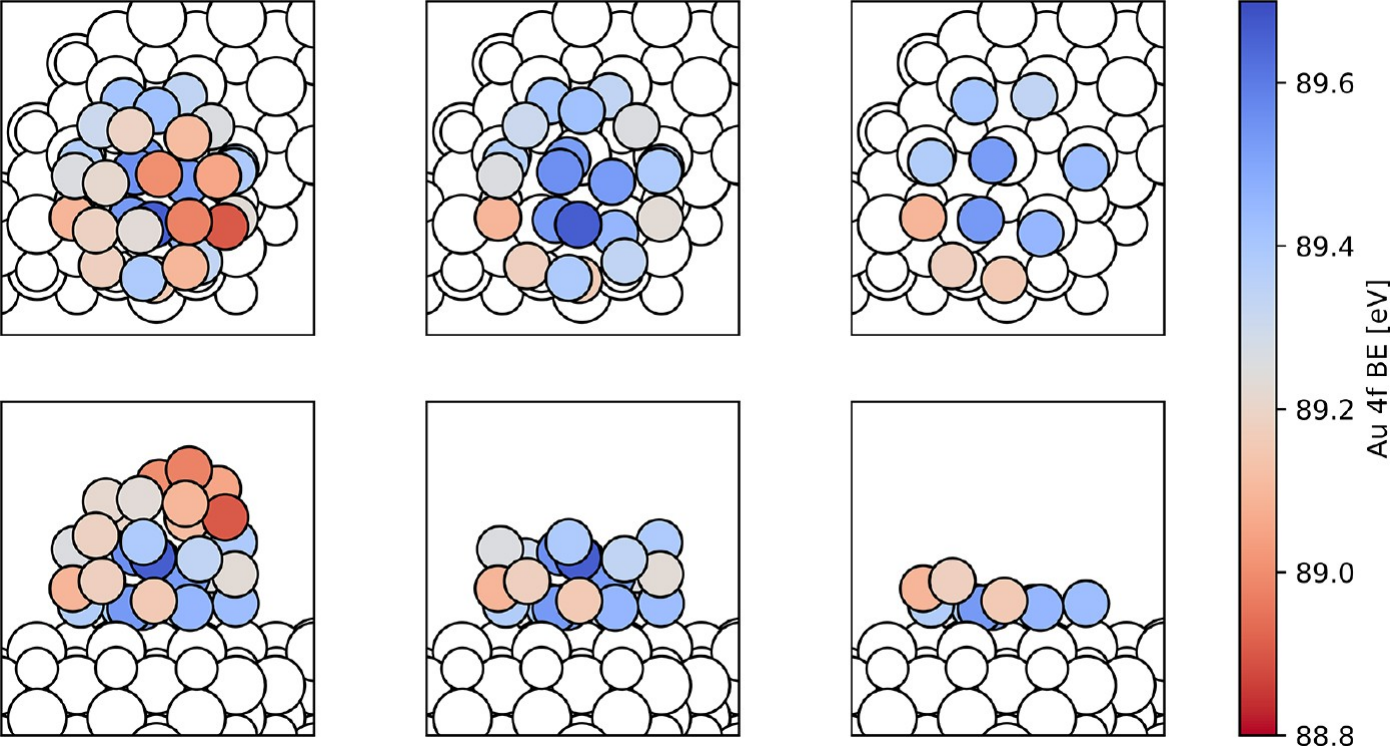
Calculated Au 4f BEs for the different atoms of the most stable electronic state found for the stoichiometric Au_31_/CeO_2_(111) model, which corresponds to the charge distribution presented in Figure 7. Some Au atoms in the images in the middle and right columns have been made transparent for clarity.

Figure 10 shows the distribution of Au 4f BEs for the different Au atoms of the most stable electronic state of the Au_31_/CeO_2_(111) model. Atoms in contact with the ceria surface have higher calculated BEs, consistent with their more-oxidized state. In turn, Au atoms distant from the Au/ceria interface, which are more metallic or reduced, have correspondingly lower BEs and are closer to BEs of metallic systems. This is consistent with the assignments of the XPS peaks to Au^0^ and Au^+^, which identified the latter as interface Au atoms in direct contact with the ceria surface.

**Figure 11:**
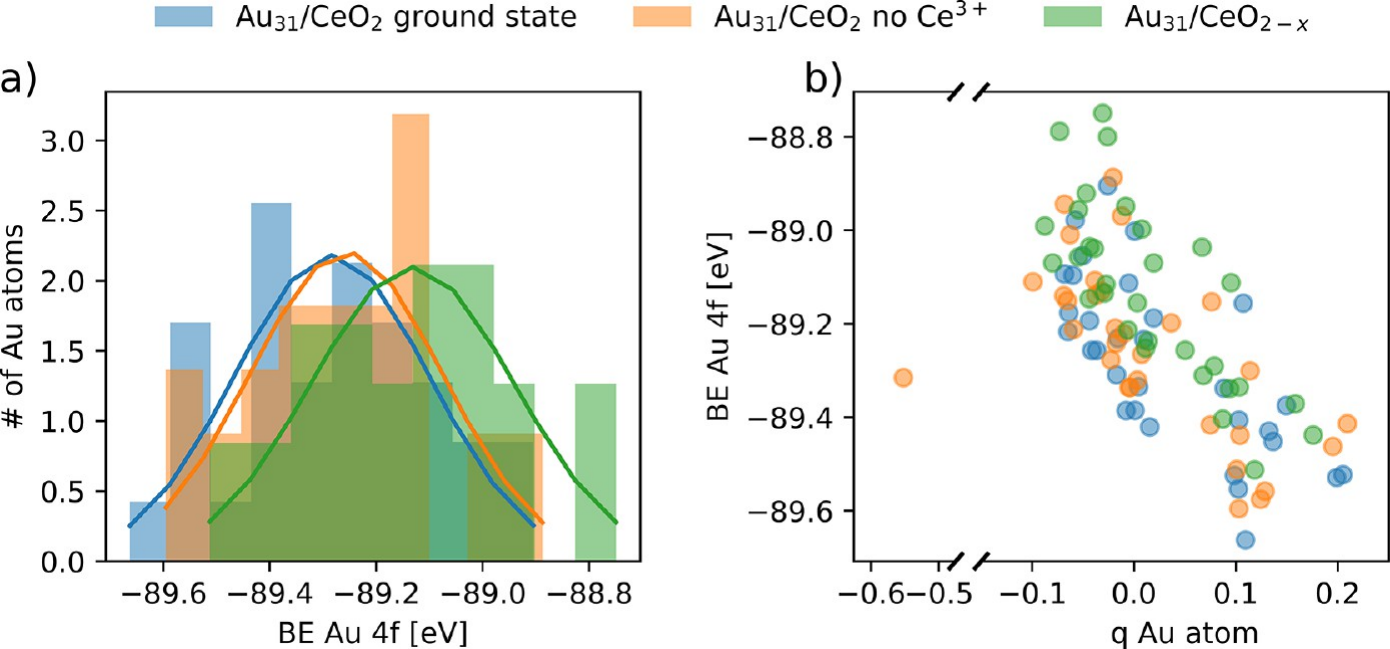
(a) Distribution of calculated Au 4f BEs for the atoms in three different ceria-supported Au_31_ particle states. Blue bars correspond to the most stable state found for the Au_31_ particles supported on stoichiometric ceria, which corresponds to the charge distribution shown in Figure 7, featuring three electrons transferred from Au_31_ to Ce^4+^ cations; green bars correspond to an unstable electronic state for the Au particle supported on stoichiometric ceria with no electrons transferred from the Au particle to Ce^4+^; and orange bars correspond to one of the most stable states found for the Au particle supported on the partially reduced ceria surface, with one Au atom near the O vacancy with a strong negative charge. Each distribution has been fitted to a Gaussian function to estimate an approximate peak position. (b) Correlation between atomic charge of each Au atom and their calculated Au 4f binding energy.

Figure 11a shows the distribution of calculated Au 4f BEs for the different ceria-supported Au particles. We remind the reader that absolute values of the Au 4f BEs are not described well by the approach used (computed values are ∼5 eV higher than the experimental ones for 4f_7/2_ or ∼1 eV, with respect to experimental 4f_5/2_), and we, therefore, focus on relative shifts instead. The distributions were fitted with a Gaussian function to estimate peak positions. It appears that the resulting differences in peak positions are much smaller than the spread in BE values calculated for each individual state. In fact, the calculated BEs correlate nicely with the atomic charge of the Au atoms, which is shown on the right plot of Figure 11b. Importantly, the notably negatively charged Au atom does not have a characteristic low BE, which hinders the detection of its formation by XPS. Moreover, the relative contribution of such Au^δ−^ species to the overall XPS spectra is expected to be low, because their formation requires direct contact between an Au atom and a surface oxygen vacancy.

The shift in peak positions calculated for Au particles reduced either by the formation of the vacancy–Au^δ−^ pair or by inducing an electronic state without Ce^3+^ cations is consistent with the shifts measured experimentally for the partially reduced model samples. This substantiates the interpretation that the partial reduction of ceria results in electron transfer to the supported Au particles.

### 3.2 Au/CeO_2_ Catalyst Exposed to Reactants

In previous sections, we addressed the effects of preparation conditions and support partial reduction on the electronic structure and size of ceria-supported Au NPs under ultrahigh vacuum (UHV) conditions. Exposure to reactants affects the structure and oxidation states of both reducible oxides and metal NPs.^90,91^ For instance, according to ab initio molecular dynamics simulations, a sufficiently high CO coverage on Au NPs deposited on ceria can result in a reversible detachment of single Au atomic species from the NP as Au–CO complexes anchored to the support.^20^ Therefore, understanding the effects of the MSI on the catalytic properties of the ceria-supported Au particles requires studying these systems under more-realistic conditions resembling operando regimes. To do this, we performed NAP-XPS experiments, which allow one to follow the evolution of the electronic structure of the Au/CeO_2_ system exposed to different reactive environments. We relate the changes in electronic structure (or lack thereof) to CO vibrational peaks from DRIFTS experiments and support their interpretation with DFT calculations.

#### 3.2.1 Evolution of Au and Ce Oxidation States upon Exposure to O_2_ and CO: NAP-XPS Study

We performed the NAP-XPS experiments by exposing samples to pressures up to 1.4 mbar of CO or O_2_ at various temperatures. This allows us to evaluate how reductive or oxidative environments affect the oxidation states of the Au/CeO_2_ systems. We focus our attention on sample 1 at elevated pressures of CO or O_2_ and sample 5 exposed to CO. We investigated the samples at a temperature representative of reaction conditions with high CO oxidation activity (∼300 °C). This temperature is high enough to enable efficient bulk diffusion of oxygen in CeO_2_,^92^ which ensures the formation of O vacancies both in the vicinity of deposited Au NPs and within the mixed Au–CeO_2_ layer.

**Figure 12:**
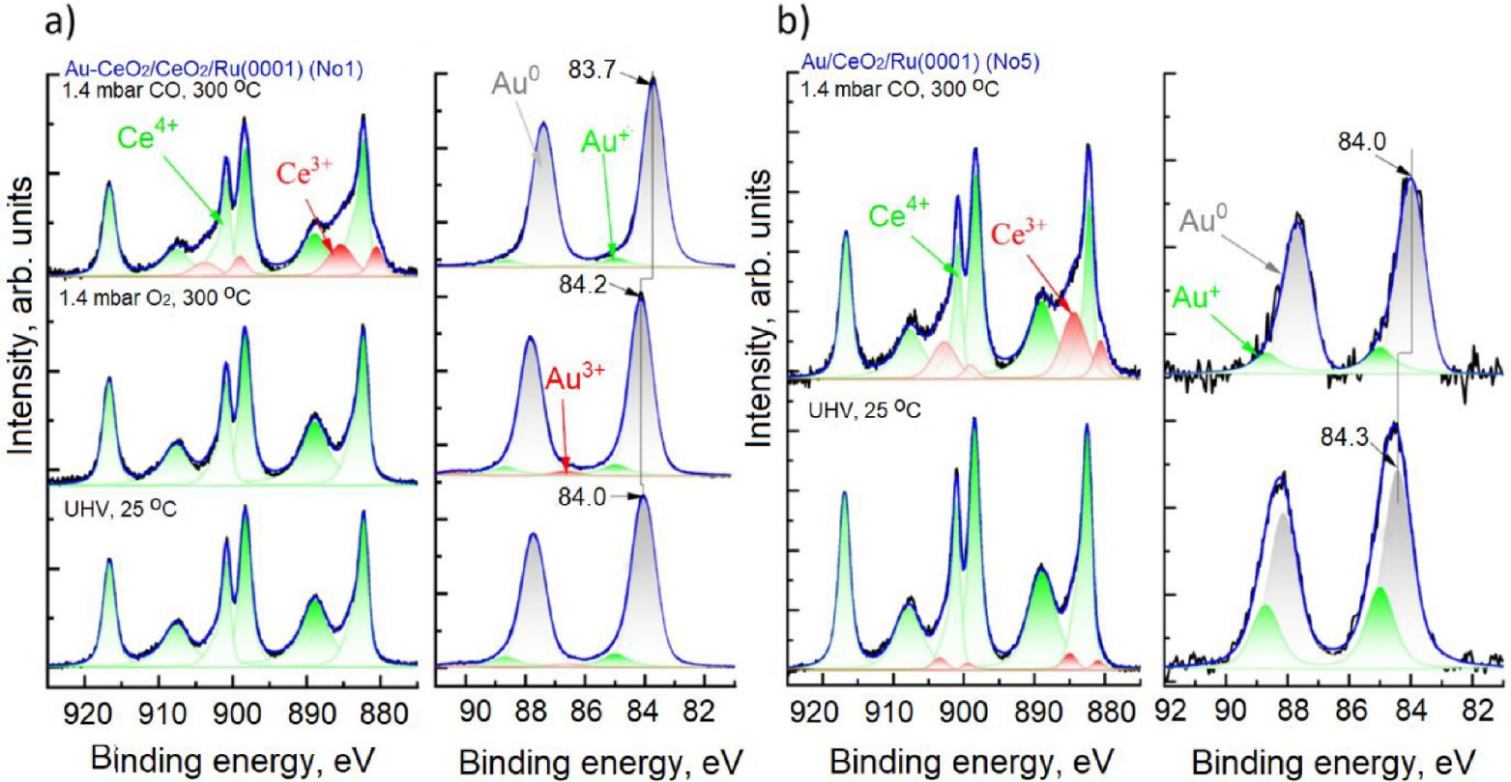
(a) Ce 3d and Au 4f spectra acquired from Au–CeO_2_/CeO_2_/Ru(0001) sample (sample 1) in UHV and the presence of O_2_ and CO at 300 °C, respectively (left panels). (b) Ce 3d and Au 4f spectra acquired from Au/CeO_2_/Ru(0001) sample (sample 5) in UHV and the presence of CO at 300 °C, respectively (right panels). Ce 3d: recorded spectra (black), three Ce^4+^-related doublets (green), two Ce^3+^-related doublets (red), and a sum (blue) are shown. Au 4f regions: measured spectra (black), their fitting in Au^3+^ (red), Au^+^ (green), Au^0^ (gray), and a sum (blue) are also shown.

Figure 12 shows Ce 3d and Au 4f NAP-XPS spectra collected in the presence of O_2_ and CO. The reference UHV spectra are also shown. Exposure of Au–CeO_2_/CeO_2_/Ru(0001) (sample 1) to 1.4 mbar of O_2_ at 300 °C had a minimal effect on the oxidation states of Au and Ce. However, it led to a 0.2 eV shift of the Au^0^ 4f_7/2_ peak position toward higher BE and a slight decrease in Ce^3+^ concentration (see Table 3). According to our interpretation of the UHV-XPS data, the shift, in the absence of an apparent increase of the Au^+^ peak, indicates that O vacancies at the Au/ceria interface are healed, leading to the transfer of electrons from Au NPs back to the ceria. However, we cannot fully discard the fact that the observed Au^0^ 4f_7/2_ peak shift is due to chemisorbed O_2_, which happens more favorably on small Au particles^93,94^ than on extended surfaces,^29^ and which has been observed for other oxide-supported Au NP catalysts.^30^ Nevertheless, the fact that exposure to O_2_ does not significantly change the concentration of Au^+^ suggests that the direct oxidation of Au particles by O_2_ does not occur under such conditions. The subsequent exposure of the sample to 1.4 mbar of CO at 300 °C shifted the Au^0^ 4f_7/2_ peak position by 0.3 eV to lower BE. The contribution of Au^+^ states to the total Au 4f signal remained similar, while the Au^3+^ ions completely disappeared, most probably because of their low thermal stability. Another noticeable effect of exposure to CO was the partial reduction of the ceria substrate. This suggests that CO oxidation on such mixed Au–CeO_2_ sample at 300 °C involves the MvK mechanism where CO adsorbed on Au particles is oxidized by O atoms from the ceria lattice.

**Table 3:** Summary of UHV and NAP-XPS Study of the Oxidation States of Ce and Au in the Au–CeO_2_/CeO_2_/Ru(0001) and Au/CeO_2_/Ru(0001) Model System^a^

		Ce 3d		Au 4f			
sample	measurement conditions	Ce^3+^ (%)	Ce^4+^ (%)	Au^0^ (%)	Au^+^ (%)	Au^3+^ (%)	Au^0^ 4f_7/2_ peak position (eV)
No. 1:Au–CeO_2_/CeO_2_/Ru(0001)	UHV, 25 °C	<1	99	87	11	2	84.0
	1.4 mbar O_2_, 300 °C	<1	99	87	10	3	84.2
	1.4 mbar CO, 300 °C	18	82	92	8	<1	83.7
No. 5: Au/CeO_2_/Ru(0001)	UHV, 25 °C	<1	99	67	33	<1	84.2
	1.4 mbar CO, 300 °C	21	79	83	17	<1	84.0

a Relative contributions of the fitted components are calculated as peak areas.

In the case of sample 5 (Figure 12), the exposure to CO at 300 °C also resulted in a partial reduction of ceria and a shift of the Au^0^ component in the Au 4f spectrum by 0.2 eV to lower BE. In addition, the signal from Au^+^ decreased, which can be attributed to the growth of Au NPs at elevated temperatures. As discussed above, Au^+^ species are located at the Au/CeO_2_ interface, whose area relative to the volume of the particle decreases as the Au particles grow.

The NAP-XPS measurements, thus, clearly show that the formation of oxygen vacancies in the vicinity of Au particles results in the charge transfer from the ceria support to the Au particle, also in the presence of CO.

#### 3.2.2 Evaluation of CO Adsorption Sites: In Situ DRIFTS

**Figure 13:**
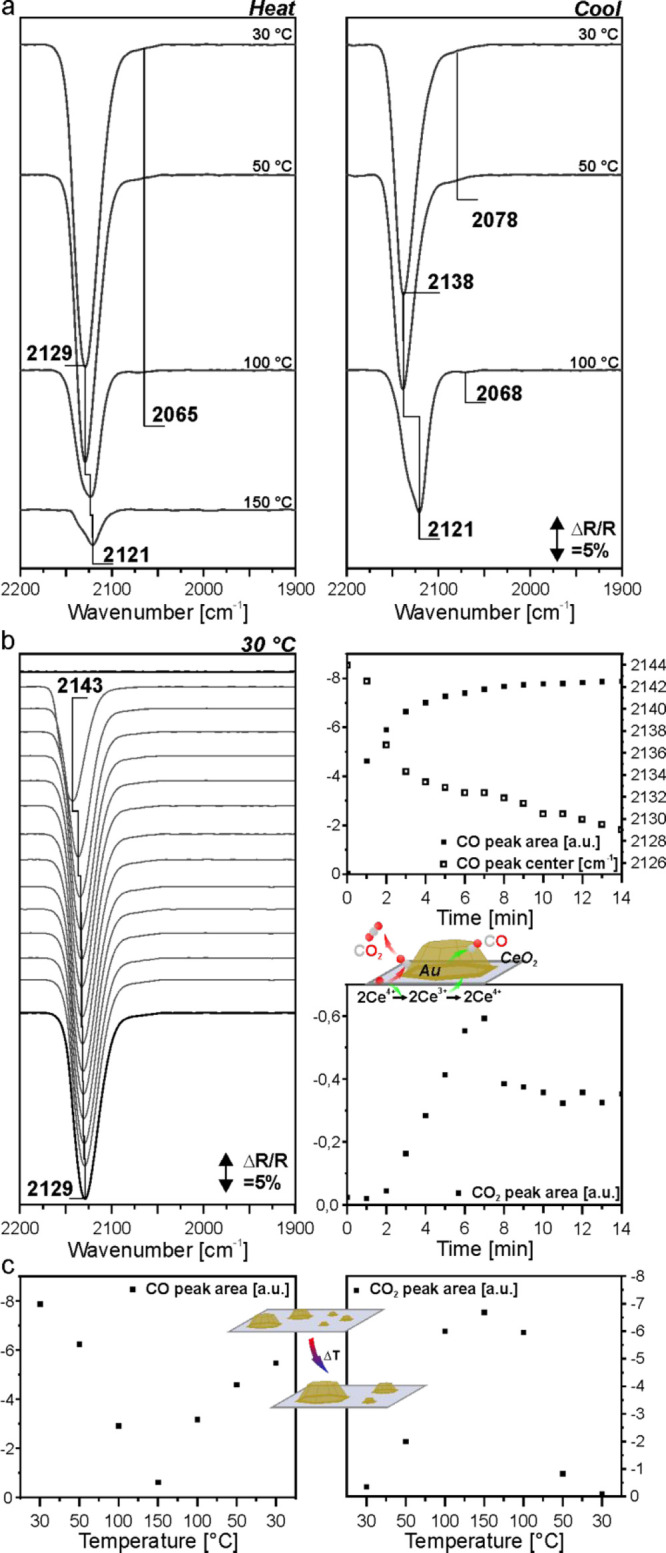
(a) The last spectra in CO during heating and cooling of Au/CeO_2_; (b) time-resolved spectra during CO dosing and the corresponding integration results; (c) comparison of CO peak area and CO_2_ peak area at different temperatures.

We applied in situ DRIFTS to investigate the samples under conditions resembling those of the CO oxidation reaction (Figure 13). Here, CO also serves as a probe molecule to scrutinize the nature and abundance of various adsorption sites. We use CeO_2_ nanooctahedra as support material, since their surface is dominated by (111) planes similar to the ceria surface of the model systems investigated by NAP-XPS.^95^

According to the literature, Au atoms in CeO_2_-supported Au NPs can be in the metallic state, or (slightly) negatively/positively charged. The charge state is dependent on the size of the Au NPs, the nature of the support, and the specific location in the NP.^33,43,96^ Since the charge density of the metal-binding partner directly influences the frequency of the CO adsorbate, different regions of the CO spectra on Au particles have been previously assigned to CO adsorbed on Au^δ+^ (>2140 cm^–1^), Au^0^ (∼2115 cm^–1^), and Au^δ−^ (<2100 cm^–1^) sites.^33,34,96,97^ Note that the respective spectral regions overlap to some extent, and the CO peak position shifts to lower wavenumbers with increasing electron density at the Au center. This is rationalized by the back-donation of electron density from Au to CO in the Blyholder model,^33^ where the electron density is transferred to the antibonding 2π* orbital of CO. This increases the strength of the CO–Au bond and concomitantly weakens the C–O bond. Thus, a shift of the peak position to lower wavenumbers is observed.^98^ CO adsorption on Au NPs occurs at low-coordinated sites only, but not on flat terraces.^99,100^ As a result, the CO coverage on the Au NPs is generally rather low. Hence, static and dynamic CO coupling effects are relatively weak in comparison to CO on other transition metals. Regarding the Au coordination and CO coverage, Moskaleva and co-workers found similar adsorption properties of 6- and 7-coordinated Au sites and shifts to lower frequencies upon increasing CO coverage at undercoordinated sites of stepped Au surfaces.^101^ However, opposite shifts were observed when increasing the CO coverage on other metals.^102^ The authors also found that prolonged exposure to CO leads to reconstruction of the Au stepped surface.

Because of these findings, we anticipate multiple contributions in the experimental CO–DRIFTS spectra. To support the assignment of the CO adsorbate signals, we performed additional DFT calculations of CO adsorption on the Au_31_/CeO_2_(111) structural model.

**Figure 14:**
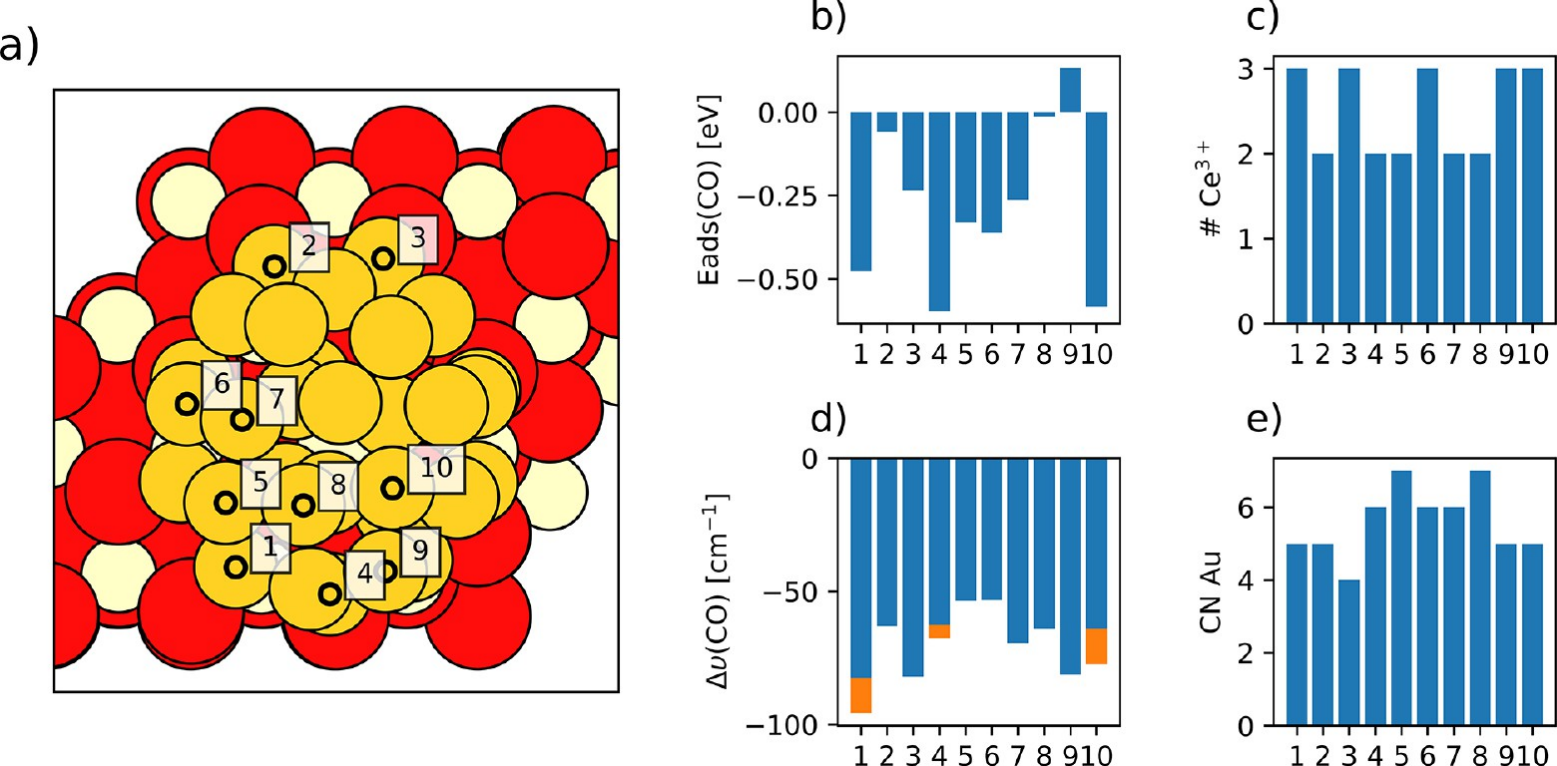
Evaluated Au adsorption sites and related calculated properties for CO adsorption on the Au_31_/CeO_2_(111) model. Circles in the structure image (panel (a)) indicate the position for each sampled Au atom, which are labeled from 1 to 10. Surface O, Ce, and Au atoms are represented by bright red, beige, and yellow circles, respectively. (b) CO adsorption energies, (c) the number of Ce^3+^ cations formed, (d) the CO vibrational stretching frequency, and (e) the coordination number of the Au atom are also plotted for the different positions. Orange bars in panel (d) correspond to frequencies calculated for CO adsorbed on Au_31_/CeO_2_(111) in the electronic state without any electrons transferred to Ce^4+^ cations (i.e., resulting in a more reduced Au particle).

In particular, we calculated the adsorption energies and corresponding vibrational frequencies of CO adsorbed in several inequivalent positions of the ceria-supported Au_31_ particle by placing a single CO molecule on 10 different sites (depicted in Figure 14a) of the Au_31_ particle, including atoms in contact with the ceria surface and atoms from different layers of the NP, as well as atoms with coordination numbers from 4 to 7. Thus, we do not evaluate all CO adsorption sites available at the NP surface, but only selected ones representing the variability in coordination and distance from the metal/oxide interface. To determine the effect of the Au particle charge on the adsorption properties, we considered the adsorption of CO on both the most stable electronic state found, with three electrons transferred from the Au_31_ particle to the ceria substrate (shown in Figure 7) and on a state without any electrons transferred, representing less oxidized Au particles. All states obtained with adsorbed CO contain either two or three Ce^3+^, which indicates that the adsorption of CO does not notably change the number of electrons transferred from the Au NP to the support. Nevertheless, we reiterate that a more thorough sampling of electronic states might lead to a more stable distribution of these two or three Ce 4f electrons (Figure 14c), as was the case for the Au_31_/CeO_2_(111) system without adsorbed CO, vide supra.

CO adsorption energy values *E*_ads_(CO) range from −0.61 eV to +0.12 eV (Figure 14b), with the most stable (favorable) adsorption site corresponding to Au atom labeled 4 with coordination number 4 and two Ce^3+^ cations present in the model. It is followed in terms of stability by adsorption sites 10 and 1, with *E*_ads_(CO) values of −0.60 eV and −0.49 eV, respectively, both with 3 Ce^3+^ cations. Adsorption on Au atoms directly in contact with the ceria surface generally is significantly less stable. For example, adsorption on sites 2 and 3 is characterized by *E*_ads_(CO) values of −0.07 and −0.25 eV, respectively. However, such weak adsorption is not only due to the particularly high coordination of these sites (the corresponding coordination numbers being 5 and 4, see Figure 14e). In fact, the calculated adsorption energy values *E*_ads_(CO) seem to be poorly correlated to the coordination numbers of the Au site (see Figure S6 in the ESI). However, the adsorption energies of each adsorption site are sensitive to the number and position of reduced Ce^3+^ cations, as well as to the symmetry of the occupied f orbital and the total occupation value. The orbital occupation should ideally be as close to unity as possible since the +U correction penalizes departures from idempotency, but the calculation sometimes converges to less-stable states with occupation values of ∼0.6 or 0.7.

Therefore, the multitude of factors affecting the overall stability of the CO/Au_31_/CeO_2_(111) system makes it difficult to precisely quantify the bonding properties between CO and Au based on the *E*_ads_(CO) values alone. Calculated CO vibrational frequencies for different adsorption sites appear to be more consistent complementary information for characterizing the nature of the Au–CO interaction, since the frequencies are mainly dependent on the local properties of the adsorption site. CO stretching frequency shifts calculated with respect to the frequency of a gas-phase CO molecule are shown in Figure 14d. These range from −82 cm^–1^ to −53 cm^–1^, which correspond to experimental frequencies of 2061 cm^–1^ and 2090 cm^–1^, respectively. According to literature assignments,^33^ these frequencies correspond to CO adsorbed on negatively charged Au, whereas frequencies calculated for undercoordinated sites on stepped Au surfaces range from 2011 cm^–1^ to 2054 cm^–1^.^101^ However, our calculations indicate that CO does not bind to the negatively charged Au atoms formed close to O vacancies. This is consistent with the very low adsorption energies calculated by Wang et al. for CO on single-atom Au^–^ sites anchored on O vacancies of partially reduced ceria.^20^ These low adsorption energies may also explain the deactivation of perimeter (interface) sites of ceria-supported Au particles upon partial reduction of the oxide substrate reported by López-Haro et al.,^22^ which these authors attributed to structural strain rather than the formation of negatively charged Au species.

**Figure 15:**
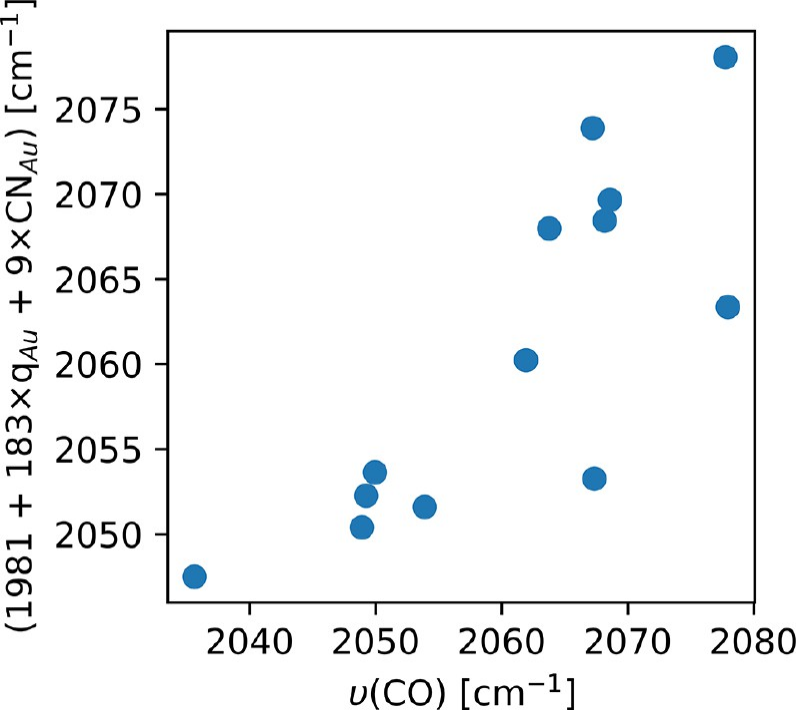
Quality of the fit of the calculated vibrational frequencies ν(CO) [cm^–1^] for CO adsorbed on Au_31_/CeO_2_(111) as a function of the atomic charge *q*_Au_ of the Au atom after binding to CO and its coordination number (CN_Au_): ν(CO) = 1981 + 183*q*_Au_ + 9CN_Au_. The correlation coefficient of the fit is 0.81.

Unlike what was found for the adsorption energies, there is some correlation between the calculated CO stretching frequency and coordination number of the adsorption site (with a correlation coefficient of 0.70; see Figure S7 in the ESI), with lower coordination numbers leading to lower stretching frequencies. There is also a slight correlation between the CO stretching frequency and the charge of the Au atom after its binding to CO (with a correlation coefficient of 0.56), with more positive charges leading to higher frequencies. We fit a simple linear equation describing the frequency as a function of the Au atomic charge and coordination number, with a correlation coefficient of 0.81 (see Figure 15). This regression is far from providing an accurate prediction of the vibrational frequency, but it does reveal a trend that is helpful for interpreting the experimental DRIFTS results presented above. We note that the Au atoms binding CO become more oxidized upon CO adsorption, exhibiting positive Bader charges in all cases.

We investigated with DRIFTS the samples at temperatures for which the CO oxidation activity is low (30 and 50 °C) and high (100 and 150 °C). Note that the CO partial pressure applied during the DRIFTS experiments is 1 order of magnitude higher than those used during the NAP-XPS measurements (50 mbar and 1.4 mbar, respectively). Therefore, the samples were reduced more significantly during the in situ DRIFTS measurements. First, we investigated pure CeO_2_ nanooctahedra as a reference sample. The spectra recorded during exposure to CO at different temperatures are displayed in Figure S8 in the ESI. The absence of CO adsorbate bands for the Au-free sample indicates that all CO peaks observed on the Au/CeO_2_ sample are associated with the Au NPs. In Figure 13a, the last spectra under exposure to CO are displayed at the respective temperature steps for the Au/CeO_2_ sample. The spectra are dominated by a peak in the wavenumber range from 2121 cm^–1^ to 2138 cm^–1^. In addition, there is a small shoulder present at <2100 cm^–1^. Generally, the peaks decrease reversibly upon annealing. There is no peak corresponding to carbonate species, in contrast to species identified by Bernal, Calvino, and co-workers.^103,104^ This is probably due to the fast desorption of such carbonate species as CO_2_ at the applied conditions.

Comparing the peak positions with the DFT results, we acknowledge that there is no perfect correspondence between the calculated and measured frequencies in terms of absolute numbers. However, the clear trends derived from the calculations allow us to rationalize the experimental peak positions and their evolution. Considering the calculated data presented above, we assign the major peak (2121–2138 cm^–1^) to CO adsorbed on slightly positively charged (Au^δ+^) and metallic (Au^0^) atoms. We also tentatively assign the weak shoulder at <2100 cm^–1^ to CO adsorbates on low-coordinated Au sites with a small positive charge, in contrast to the previous assignment of this spectral feature to CO on Au^δ−^.^33^

Generally, the peaks decrease reversibly upon annealing and the position of the major peak shifts to lower wavenumbers (see Figure 13a), whereas it shifts to higher wavenumbers upon cooling to room temperature again. To elucidate the origin of this trend, the effect is analyzed as a function of time. Exemplarily, we focus on the measurement at 30 °C (left panel in Figure 13b), but a similar behavior is observed at the other temperatures as well (see Figure S9 in the ESI). The top spectrum was recorded in an Ar atmosphere, and a CO peak starts to evolve at 2143 cm^–1^ upon CO dosing. It grows in intensity and undergoes a red-shift to 2129 cm^–1^ during CO dosing. This is more clearly reflected in the top right panel in Figure 13b, showing an increase in the integrated peak areas and a peak shift to lower wavenumbers. This observation supports our assumption that coupling between the CO molecules is negligible. Note that we would expect a shift to higher wavenumbers with increasing CO coverage on other transition-metal surfaces, such as Pd or Pt, where coupling effects occur.^102^

The respective CO peak shift coincides with the progressive oxidation of CO and partial reduction of the ceria substrate, as evidenced by the corresponding integrated peak areas for the formed CO_2_ (Figure 13b, lower right). Thus, we explain these observations as follows: partial reduction of the ceria substrate upon CO oxidation and subsequent reoxidation results in an electron transfer to the Au NPs. This electron transfer increases the back-bonding from the Au NPs to CO and leads to a red-shift of the CO stretching mode (as observed in the time- and temperature-resolved spectra). Following this argument, enhanced CO oxidation causes a stronger partial reduction of the ceria support, which eventually leads to a stronger red-shift. Note that Au/CeO_2_ is most active for CO oxidation at 100 and 150 °C, which agrees with the evolution of the CO peak position when the peak reaches the lowest wavenumber (2121 cm^–1^).

The correlation found between the calculated CO stretching frequencies and atomic charges of the Au binding sites supports the assignment of the peak shifts observed during the DRIFTS experiments upon partial ceria reduction to electron transfer to supported Au particles. We further evaluated this effect by calculating the frequencies of CO adsorbed on positions 1, 4, and 10 for the electronic state without any electrons transferred to Ce^4+^ cations (i.e., resulting in a more-reduced Au particle), revealing a consistent shift of the C–O frequencies to lower wavenumbers by 13, 4, and 13 cm^–1^, respectively (see orange bars in Figure 14d). Coverage-related effects, as suggested by previous work on stepped surfaces,^101^ are, in turn, discarded, because of the evolution of the shifts with temperature. In particular, the major peak shifts to lower wavenumbers upon increases in temperature at which CO coverage is lower (see Figure 13c).

The peak shift to lower wavenumbers upon annealing and the reverse behavior upon cooling is dependent on the experimental procedure. The intermediate oxidative treatment ensures full reoxidation of the support at each temperature step. Thus, we probe the partial reduction of the support at the respective temperature step instead of a progressive temperature-programmed reduction. Interestingly, the peak is located at even higher wavenumbers after the cooling ramp, compared to the start of the experiment (2129 cm^–1^ vs 2138 cm^–1^). We attribute this effect to structural changes of the Au NPs during the procedure, which affect the CO oxidation activity.

We use the CO peak area as an indicator for changes in size and morphology of the Au NPs.^100^ In the left panel in Figure 13c, the integrated peak area is shown for the last spectrum in CO at each temperature step. The peak area decreases upon annealing and increases again upon cooling. We attribute the decrease of the CO peak area upon heating to partial desorption of CO at elevated temperatures. Consistently, the CO peak area increases again upon cooling. However, the CO peak area is smaller at the end of the experiment as compared to the beginning (−5.5 arbitrary units vs −8 arbitrary units). This is most likely due to the sintering or restructuring of the Au NPs. Larger particles or particles with better-ordered terraces expose fewer low-coordinated sites and, thus, the number of sites where CO adsorbs and the corresponding peak area decrease. This interpretation is also supported by the trends revealed by our DFT calculations, predicting higher wavenumbers for more coordinated Au sites such as those found in larger Au particles.

In our previous work, we observed that the CO oxidation activity is dominated by the presence of small Au NPs with a high number of low-coordinated sites.^16^ Following this finding, we analyze the temperature-dependent CO_2_ formation in the right panel in Figure 13c. The corresponding DRIFT spectra are displayed in Figure S10 in the ESI. Indeed, the CO_2_ evolution at 30 and 50 °C is lower during the cooling ramp than during the heating ramp. Therefore, the ceria support is partially reduced to a lesser extent, and fewer electrons are transferred to Au particles, which is also consistent with the differences observed in CO peak positions between the heating and cooling ramps (2129 cm^–1^ vs 2138 cm^–1^).

## 4 Conclusions

The metal–support interactions between Au NPs and ceria under CO adsorption conditions, relevant for the CO oxidation reaction, have been studied using a combination of experimental techniques (XPS, SRPES, and in situ CO DRIFTS) and DFT calculations. This approach revealed the interplay between the charge distribution of Au NPs, the oxidation state of the ceria support, and the adsorption properties of CO molecules.

On the stoichiometric CeO_2_(111) support, Au atoms in contact with the O atoms of the ceria surface are notably oxidized (Au^+^), whereas the rest of the Au atoms are neutral (Au^0^). Electrons transferred to Au upon partial reduction of the ceria support can, in turn, delocalize among the neutral Au atoms of the metal particle or form [Au^δ−^–O vacancy] complexes at the Au/ceria interface. The latter involve Au atoms with a significant negative charge (of approximately −0.6 |e|) located in close proximity to an oxygen vacancy. The presence of small amounts of additional electron density on the neutral Au atoms has clear spectroscopic signatures, with measured and calculated shifts to lower Au 4f BE and lower vibrational frequencies of the CO adsorbates. In contrast, CO does not adsorb on the strongly negatively charged Au^δ−^ sites formed at the interface, and these Au^δ−^ anions do not exhibit a characteristically low calculated Au 4f BE. This explains why we are not able to identify the formation of these significantly negatively charged Au^δ−^ species experimentally.

Therefore, the major C–O stretching DRIFTS band (2121–2138 cm^–1^) for CO adsorbed on the Au/CeO_2_ catalyst is assigned to CO adsorbed on slightly positively charged or metallic Au atoms, i.e., Au^δ+^ or Au^0^. The C–O stretching frequencies are observed to be dependent on both the charge of the Au site and its coordination number. Analysis of these dependencies allows us to attribute the band shift to lower wavenumbers upon partial reduction of the ceria support to the electron transfer to the Au particle and tentatively assign the lowest wavenumber band to CO adsorbates on low-coordinated Au centers which have a small positive charge after binding CO.

Thus, this study of the charge distribution in ceria-supported Au NPs, the observed spectroscopic properties, and the effects of the reactive environment has clarified the properties of the active sites in Au/CeO_2_ catalyst in atomic detail. Importantly, we have shown that every metal site can have a unique contribution to XPS or DRIFTS peaks, depending on the location and coordination environment of the site. As a result, the spectroscopic shifts between atoms at different positions of supported metal particles may be larger than the shifts induced by reactive treatments, pinpointing the relevance of considering the heterogeneity of different sites in studies of nanostructured catalysts.

## 5 Data Availability

The DFT data calculated in this study are available in ioChem-BD^105^ (DOI: 10.19061/iochem-bd-6-138, https://iochem-bd.bsc.es/browse/handle/100/216159).

## Supporting Information Available

Figures S1–S10 and additional descriptions of the procedure for the preparation of the model samples, the catalytic activity of Au/CeO_2_(111) in CO oxidation, additional UHV-XPS spectra, additional DFT results of the charge distribution in Au_31_/CeO_2–*x*_, and additional IR spectra (PDF)
