# Supplementary material for: Metal–Support Interaction and Charge Distribution in Ceria-Supported Au Particles Exposed to CO
Source: Chem Mater. 2022 Aug 23;34(17):7916–36. doi: 10.1021/acs.chemmater.2c01659 (PMC9476549; doi:10.1021/acs.chemmater.2c01659)
Supplement: Supplementary file 1 — cm2c01659_si_001.pdf [file cm2c01659_si_001.pdf]

Electronic Supplementary Material (ESI) for

Metal-support Interaction and Charge Distribution in Ceria-Supported Au Particles Exposed to CO

Oleksii Bezkrovnyi,[1]* Albert Bruix,[2]* Dominik Blaumeiser,[3] Lesia Piliai,[4] Simon Schötz,[3] Tanja Bauer,[3] Ivan Khalakhan,[4] Tomáš Skála,[4] Peter Matvija,[4] Piotr Kraszkiewicz,[1] Mirosława Pawlyta,[5] Mykhailo Vorokhta,[4] Iva Matolínová,[4] Jörg Libuda,[3] Konstantin M. Neyman,[2,6] and Leszek Kępiński[1]

[1] *W. Trzebiatowski Institute of Low Temperature and Structure Research, Polish Academy of Sciences, 50-422 Wroclaw, Poland*
[2] *Departament de Ciència de Materials i Química Física and Institut de Química Teòrica i Computacional (IQTCUB), Universitat de Barcelona, 08028, Barcelona, Spain*
[3] *Interface Research and Catalysis, Erlangen Center for Interface Research and Catalysis, Friedrich-Alexander Universität Erlangen-Nürnberg, Egerlandstraße 3, 91058 Erlangen, Germany*
[4] *Charles University, Faculty of Mathematics and Physics, Department of Surface and Plasma Science, V Holešovičkách 2, 18000, Prague 8, Czech Republic*
[5] *Materials Research Laboratory, Silesian University of Technology, Gliwice 44-100, Poland*
[6] *ICREA (Institució Catalana de Recerca i Estudis Avançats), 08010 Barcelona, Spain*

**Corresponding authors: o.bezkrovnyi@intibs.pl* and *abruix@ub.edu*

1. Procedure for the preparation of the model samples

For NAP-XPS study of the model samples, a single-crystal Ru(0001) disc (MaTecK) was used as a substrate for the preparation of CeO_2(111) films. The Ru(0001) substrate was cleaned by Ar^+ sputtering (300 °C, 30 min, 1 kV) with further annealing in UHV at 480 °C for 5 min until no traces of carbon (or any other contaminant) or CeO_2(111)-related signal (e.g. from residues of a CeO_2 layer from previous experiment) were found in the photoelectron spectra. A 2 nm thick epitaxial CeO_2(111) films (buffer for the samples No. 1-4 and base CeO_2(111) for the sample No. 5) were prepared by physical vapour deposition (PVD) of Ce metal (Goodfellow) from an electron-beam evaporator (Tectra) in an oxygen atmosphere ($p_{O2} = 5\times10^{-7}$ mbar) at 300 °C . Mixed Au-CeO_2 films were grown on top of the buffer by simultaneous deposition of Ce and Au metal in oxygen atmosphere ($p_{O2} = 5\times10^{-7}$ mbar) at 300 °C (No. 1), 25 °C (No. 2) and -150 °C (No. 3) for 45 min. A non-stoichiometric Au-CeO_{2-x} layer (No. 4) was deposited by simultaneous deposition of Ce and Au metal in an oxygen atmosphere ($p_{O2} = 1\times10^{-8}$ mbar) for 45 min. Sample No. 5 was prepared by deposition of Au for 3 min at 25 °C in UHV. All prepared samples were studied by NAP-XPS. The regions of interest acquired during NAP-XPS measurements were Ce 3d, Au 4f, O 1s, C 1s, and Ru 3d. They were recorded at pressures ranging from 10^{-9} (UHV) up to 1.4 mbar and temperatures ranging from -150 °C to 300 °C using a monochromated Al Kα X-ray source of high intensity.

High-resolution synchrotron-radiation photoelectron spectroscopy (SRPES) measurements were performed at the Materials Science Beamline (MSB), Elettra Synchrotron Light Facility in Trieste, Italy. The MSB, with a bending magnet source, provides synchrotron light in the energy range of 21–1000 eV. The UHV end-station (base pressure 1×10^{-10} mbar) was equipped with a multichannel electron energy analyzer (Specs Phoibos 150), LEED, an Ar^+ sputter gun, and a gas inlet system. The basic setup of the chamber includes a dual Mg/Al X-ray source. In addition, two electron-beam evaporators for Ce and Au deposition were installed. Here, a single-crystal Cu(111) disc (MaTecK) was used as a substrate for the preparation of the CeO_2(111) films. Cu(111) was cleaned by the same method as the Ru(0001) until no traces of carbon or any other contaminant were found in the photoelectron spectra. An epitaxial non-stoichiometric CeO_{2-x}(111) film was prepared in several steps (No. 7). First, the 2 nm thick CeO_2 buffer was grown by PVD of Ce metal (Goodfellow) from the e-beam evaporator (Tectra) in $p_{O2} = 5\times10^{-7}$ mbar at 300 °C. Then, 0.7 nm of metallic Ce was deposited in UHV on top of it. At the last step, such prepared sample was annealed in UHV at 630 °C for 30 min). An epitaxial stoichiometric CeO_2(111) film (No. 6) was prepared in the same way as the non-stoichiometric film with the only difference that the annealing

at 630 °C was done in p_{O2} = 5×10^{-7} mbar. The similarity in the preparation parameters ensured similarly low number of atomic steps on the surface of the stoichiometric and non-stoichiometric ceria films supported by Cu(111) single-crystals. On both samples No. 6 and 7, Au nanoparticles were deposited by PVD of Au for 20 s at 25 °C in UHV.

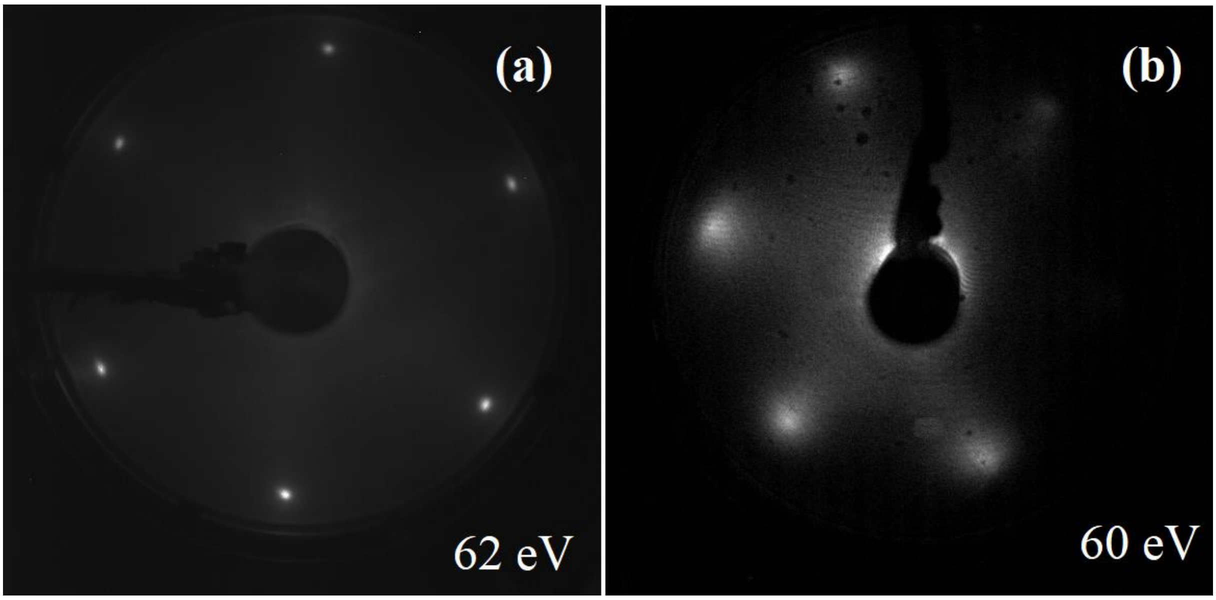

Figure S1: Typical LEED patterns of CeO_2(111) films grown on (a) Ru(0001) and (b) Cu(111).

2. Catalytic activity of $Au/CeO_2(111)$ in CO oxidation.

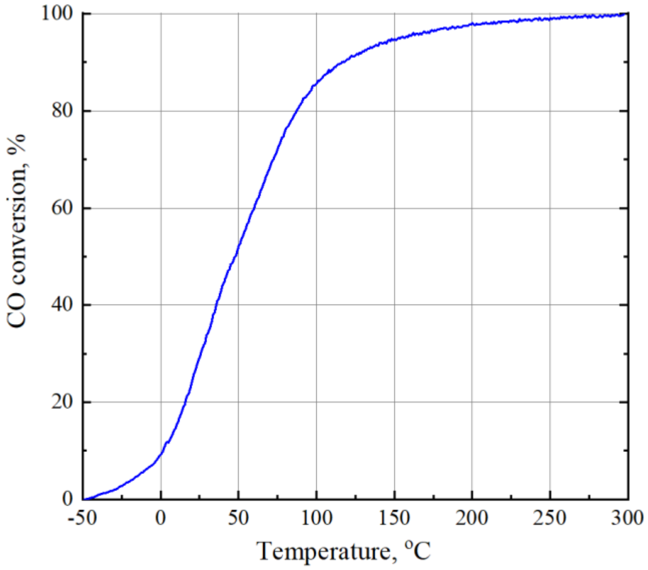

Figure S2: CO oxidation light-off plot for the powder $Au/CeO_2(111)$ sample taken from [1] depicting the catalytic activity of Au NPs supported by ceria nanooctahedra (mainly terminated by {111} faces). As seen, the process of CO oxidation begins below 0 °C, the temperature of half conversion is around 50 °C, and at 150 °C CO conversion exceeds 90 %. It agrees well with the literature data of CO oxidation on Au/CeO_2. A number of authors shows that the typical temperature window of active CO oxidation on Au/ceria powder samples, is 25…200 °C.[2–4]

3. Additional UHV-XPS spectra

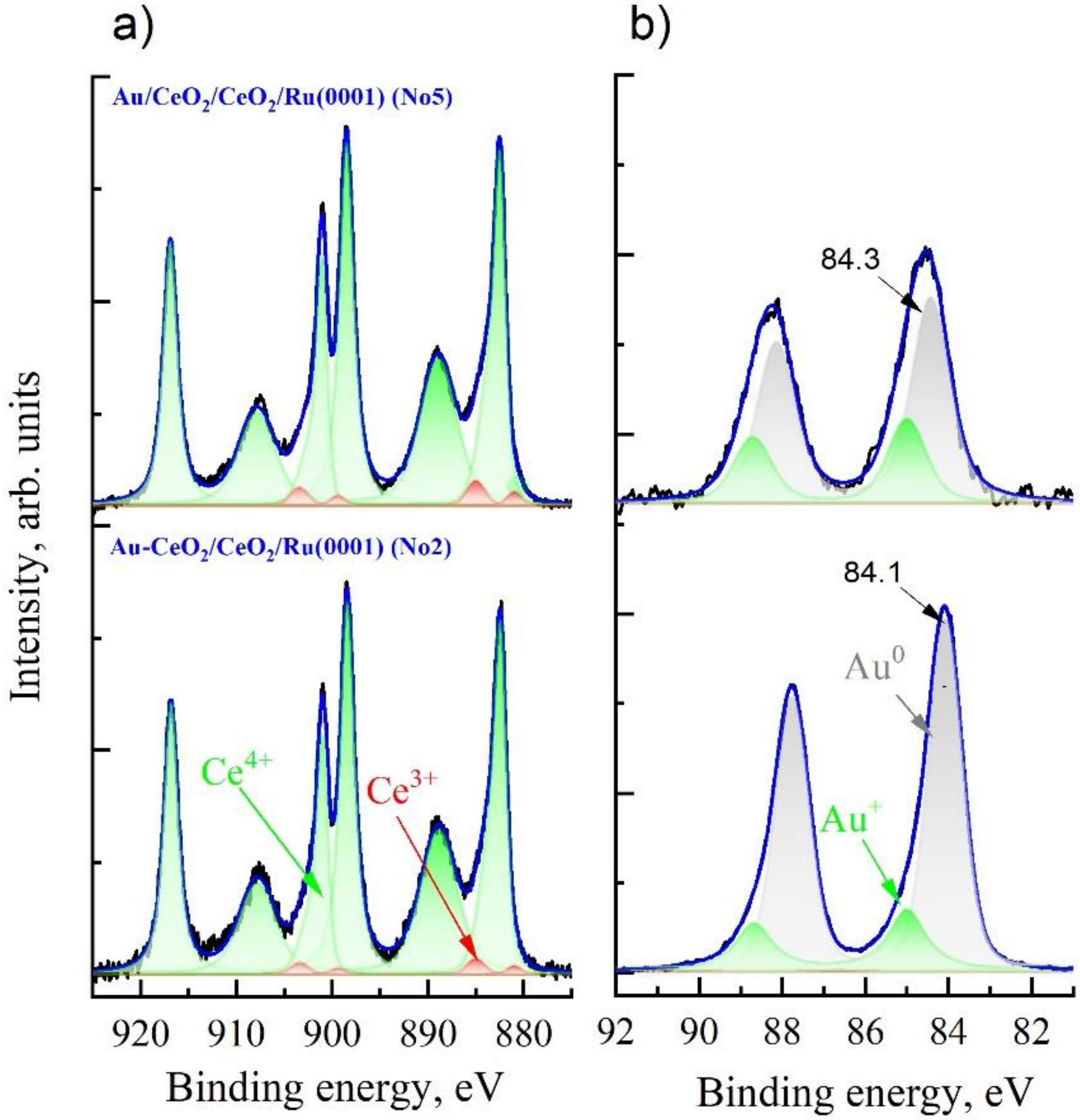

Figure S3: Normalized XPS Ce 3d (a) and Au 4f regions (b) of the UHV spectra acquired at 25 °C on the stochiometric Au-CeO_2/CeO_2/Ru(0001) (sample 2, Au-CeO_2 co-deposited at 25 °C) and Au/CeO_2/Ru(0001) (sample 5, Au deposited on CeO_2 layer at 25 °C).

4. DFT calculations of charge distribution in Au_{31}/CeO_{2-x}

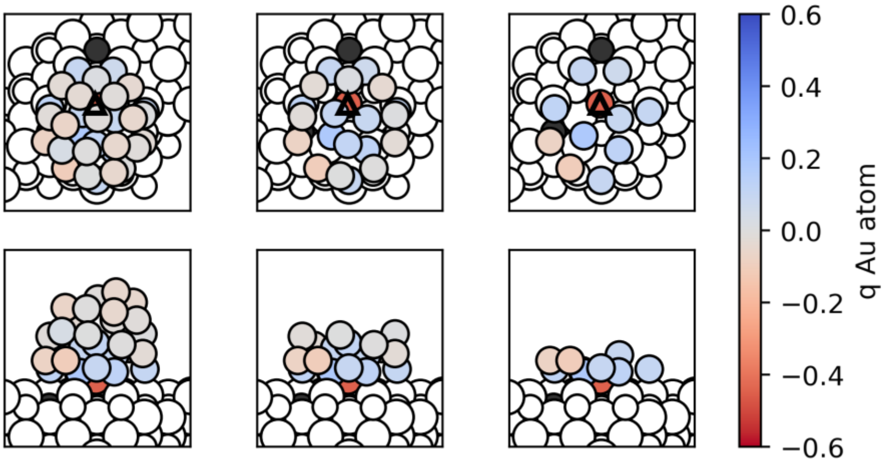

Figure S4: Top and side views of the most stable state found for the $Au_{31}/CeO_{2-x}(111)$ model (with an O vacancy in the position labeled 1 and $E_{vac}(O) = 2.60$ eV). The Au atoms are colored according to their calculated Bader charge q, and Ce^{3+} cations appear colored in black. Ce^{4+} and O atoms are shown as small and large white spheres, respectively. Some Au atoms in the images in the middle and right columns have been made transparent for clarity. The position of the vacancy is indicated with a triangle.

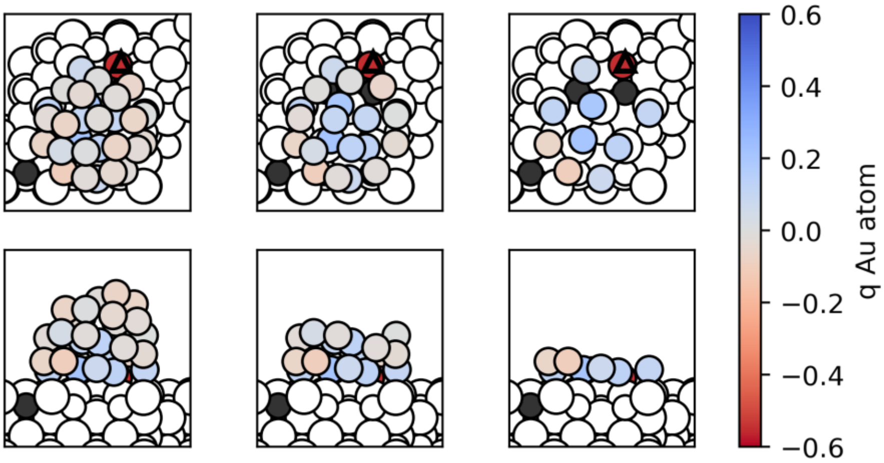

Figure S5: Top and side views of the second most stable state found for the $Au_{31}/CeO_{2-x}(111)$ model (with an O vacancy in the position labeled 5 and $E_{vac}(O) = 2.71$ eV). The Au atoms are colored according to their calculated Bader charge q, and Ce^{3+} cations appear colored in black. Ce^{4+} and O atoms are shown as small and large white spheres, respectively. Some Au atoms in the images in the middle and right columns have been made transparent for clarity. The position of the vacancy is indicated with a triangle.

5. DFT calculations of CO adsorption on Au/CeO_{2-x} catalyst

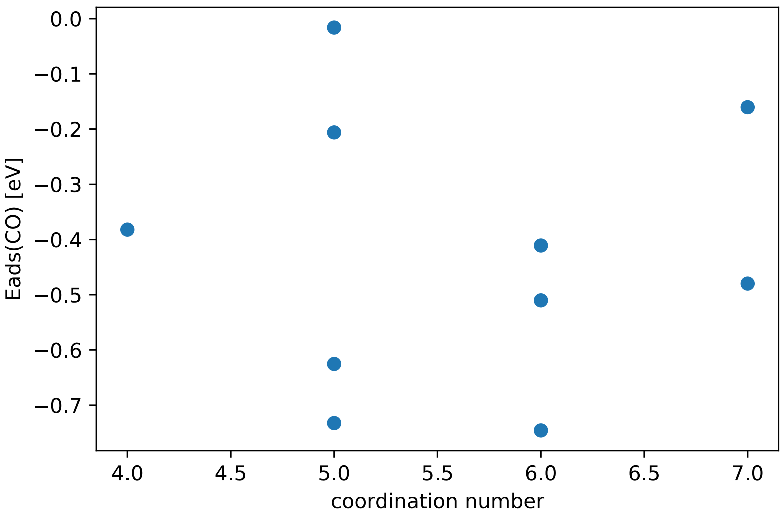

Figure S6: CO adsorption energies E_{ads}(CO) plotted vs the coordination number of the Au atom that adsorbs CO.

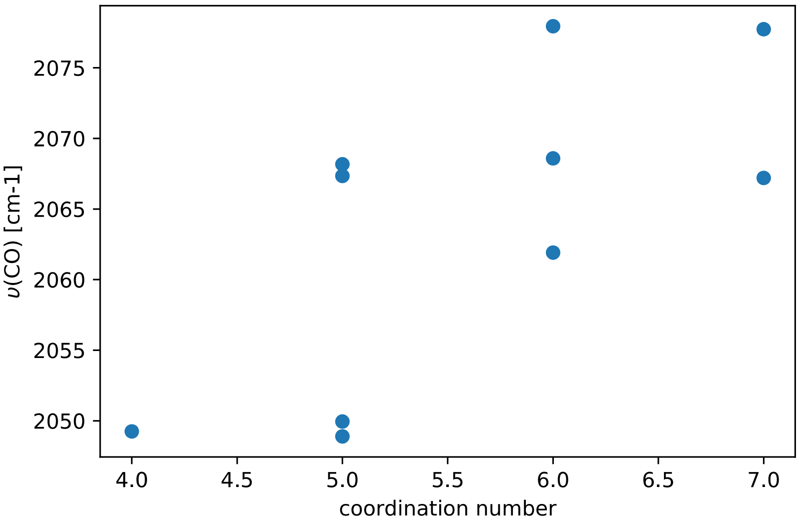

Figure S7: Calculated C-O stretching frequencies plotted vs the coordination number of the Au atom that adsorbs CO.

6. Additional DRIFTS spectra

CO adsorption on CeO_2

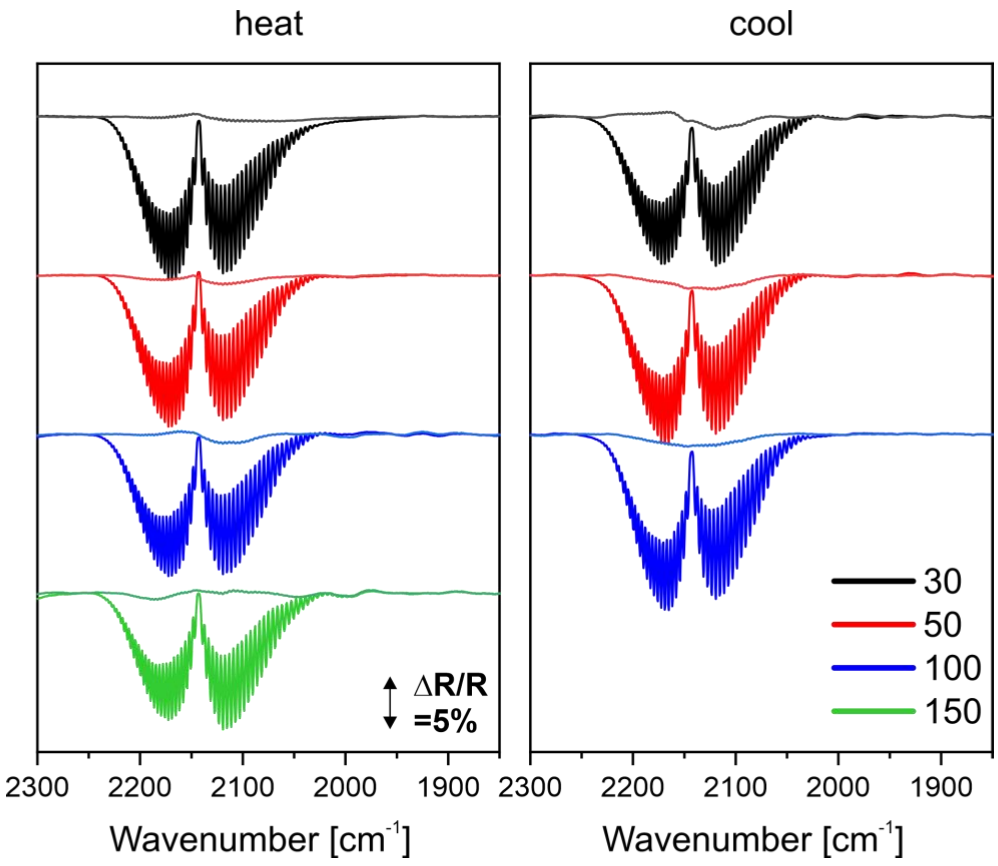

Figure S8: CO dosing on CeO_2 at different temperatures (shown in °C).

We used bare CeO_2 as a reference sample to study the CO adsorption on the support. The spectra displayed in Fig. S8 are the last ones in CO environment at the respective temperature step as well as a spectrum during evacuation. Evacuation allows removing the CO gas phase and uncovering potential CO adsorption peaks.

There are no CO adsorption peaks present in Fig. S8. Thus, all CO peaks observed on Au/CeO_2 are associated with the presence of the Au NPs.

CO dosing at different temperatures

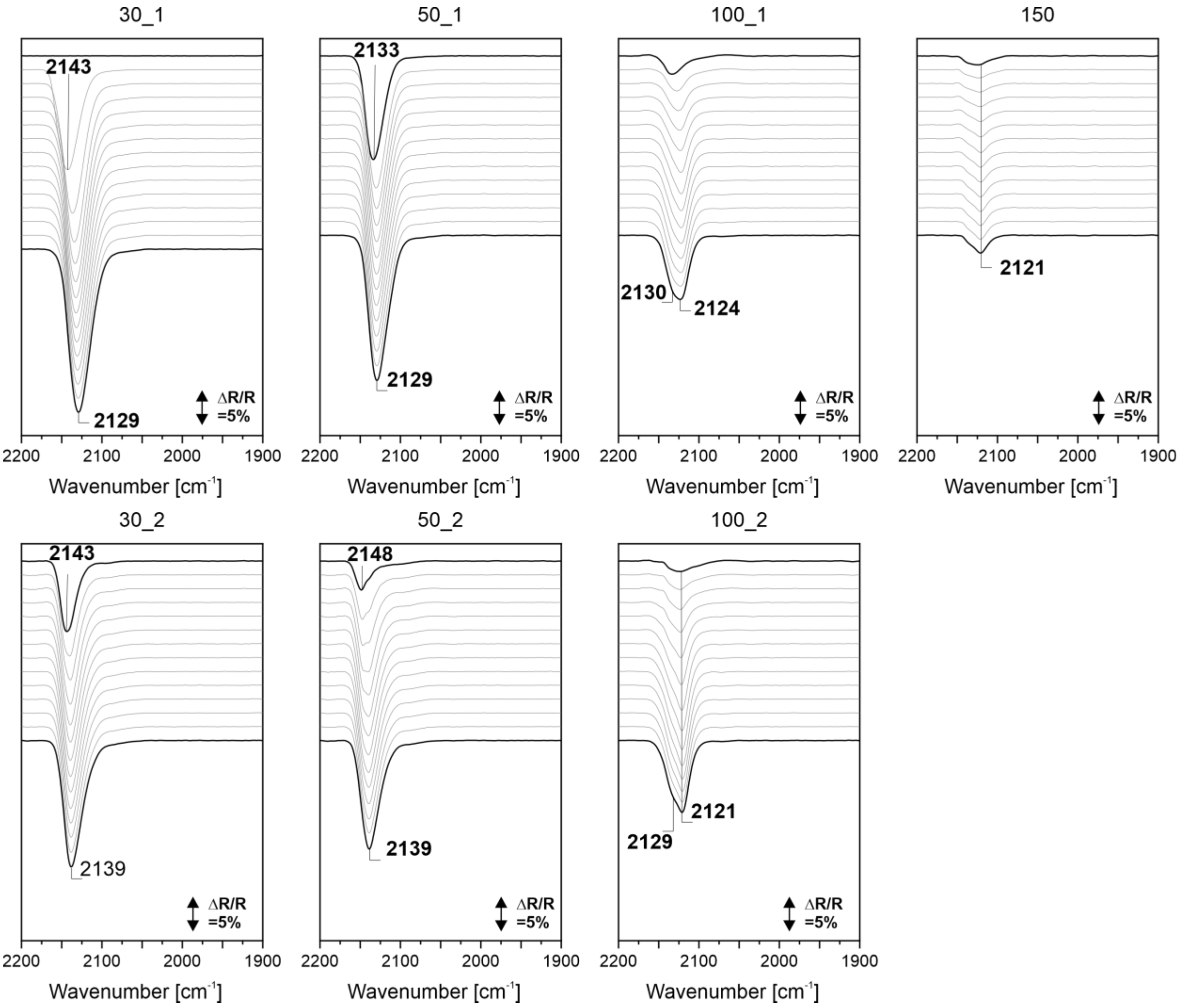

Figure S9: Time-resolved spectra during CO dosing on Au/CeO_2 at different temperatures (shown in °C) during gradual heating and cooling of the powder sample.

In Fig. S9, the dosing of CO is shown at the different temperature steps. For all temperatures, one can see that the major CO peak starts to grow in intensity but also undergoes a shift to lower wavenumbers upon dosing. As explained in the main text, we assign this to the reduction of the support upon CO oxidation. CO_2 is formed upon the reaction of lattice oxygen and adsorbed CO. This generates an oxygen vacancy. Two surplus electrons are transferred to 2 Ce^{4+} ions in the lattice. They are reduced to 2 Ce^{3+} ions, but reoxidize again upon electron transfer to Au NPs. Finally, this increases the back bonding to CO and leads to a redshift of the CO stretching vibration to lower wavenumbers.

CO_2 evolution at different temperatures

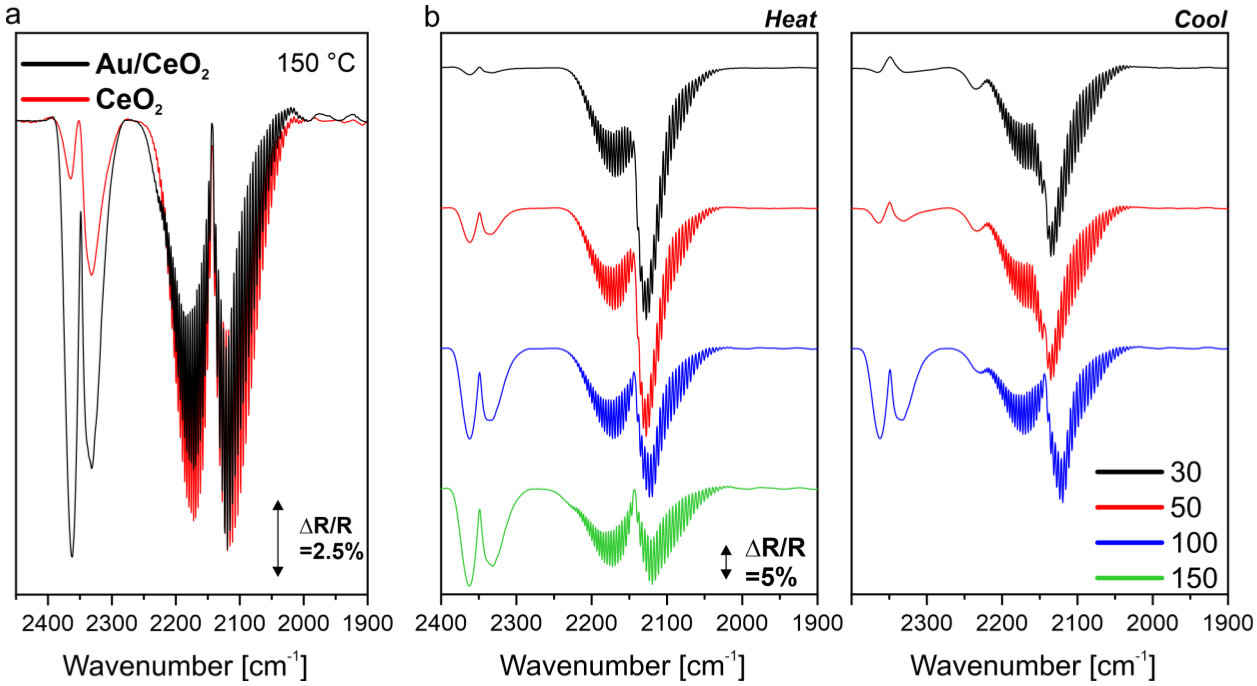

Figure S10: Different CO_2 evolution on Au/CeO_2 at different temperatures (shown in °C).

In Fig S10, the CO_2 evolution is displayed at different temperatures. Note that these spectra are not mathematically treated to remove the gas phase CO. Referring to the CO_2 signal, one can see that the CO_2 evolution is most pronounced at the highest temperatures. This is because the Au/CeO_2 sample is most active towards CO oxidation at 100 and 150 °C.

7. References

(1) Bezkrovnyi, O. S.; Kraszkiewicz, P.; Mista, W.; Kepinski, L. The Sintering of Au Nanoparticles on Flat {100}, {111} and Zigzagged {111}-Nanofacetted Structures of Ceria and Its Influence on Catalytic Activity in CO Oxidation and CO PROX. *Catal. Letters* **2021**, *151* (4), 1080–1090. https://doi.org/10.1007/s10562-020-03370-1.

(2) Trovarelli, A.; Llorca, J. Ceria Catalysts at Nanoscale : How Do Crystal Shapes Shape Catalysis ? *ACS Catal.* **2017**, *7*, 4716−4735. https://doi.org/10.1021/acscatal.7b01246.

(3) Sudarsanam, P.; Mallesham, B.; Reddy, P. S.; Großmann, D.; Grünert, W.; Reddy, B. M. Nano-Au/CeO_2 Catalysts for CO Oxidation: Influence of Dopants (Fe, La and Zr) on the Physicochemical Properties and Catalytic Activity. *Appl. Catal. B Environ.* **2014**, *144,* 900–908. https://doi.org/10.1016/j.apcatb.2013.08.035.

(4) Venezia, A. M.; Pantaleo, G.; Longo, A.; Di Carlo, G.; Casaletto, M. P.; Liotta, F. L.; Deganello, G. Relationship between Structure and CO Oxidation Activity of Ceria-Supported Gold Catalysts. *J. Phys. Chem. B* **2005**, *109* (7), 2821–2827. https://doi.org/10.1021/jp045928i.
